# Proteome-Wide Profiling of the Covalent-Druggable Cysteines with a Structure-Based Deep Graph Learning Network

**DOI:** 10.34133/2022/9873564

**Published:** 2022-07-21

**Authors:** Hongyan Du, Dejun Jiang, Junbo Gao, Xujun Zhang, Lingxiao Jiang, Yundian Zeng, Zhenxing Wu, Chao Shen, Lei Xu, Dongsheng Cao, Tingjun Hou, Peichen Pan

**Affiliations:** ^1^Innovation Institute for Artificial Intelligence in Medicine of Zhejiang University, College of Pharmaceutical Sciences, Zhejiang University, Hangzhou, 310058 Zhejiang, China; ^2^State Key Lab of CAD&CG, Zhejiang University, Hangzhou, 310058 Zhejiang, China; ^3^Institute of Bioinformatics and Medical Engineering, School of Electrical and Information Engineering, Jiangsu University of Technology, Changzhou 213001, China; ^4^Xiangya School of Pharmaceutical Sciences, Central South University, Changsha, 410004 Hunan, China

## Abstract

Covalent ligands have attracted increasing attention due to their unique advantages, such as long residence time, high selectivity, and strong binding affinity. They also show promise for targets where previous efforts to identify noncovalent small molecule inhibitors have failed. However, our limited knowledge of covalent binding sites has hindered the discovery of novel ligands. Therefore, developing in silico methods to identify covalent binding sites is highly desirable. Here, we propose DeepCoSI, the first structure-based deep graph learning model to identify ligandable covalent sites in the protein. By integrating the characterization of the binding pocket and the interactions between each cysteine and the surrounding environment, DeepCoSI achieves state-of-the-art predictive performances. The validation on two external test sets which mimic the real application scenarios shows that DeepCoSI has strong ability to distinguish ligandable sites from the others. Finally, we profiled the entire set of protein structures in the RCSB Protein Data Bank (PDB) with DeepCoSI to evaluate the ligandability of each cysteine for covalent ligand design, and made the predicted data publicly available on website.

## 1. Introduction

Large-scale scientific exploration in biomedical sciences such as genome sequencing and structural genomics has enabled us to discover many new potential drug targets [1, 2]. Validating a new candidate target for drug discovery requires the development of chemical probes to explore the consequences of perturbing the functions of the protein [3–5]. However, only a small portion of proteins have been successfully targeted by selective ligands and many proteins are even considered undruggable because of the lack of suitable binding pockets on the protein surfaces [6, 7]. The use of covalent ligands offers potential solutions to this problem, and the design and discovery of novel covalent inhibitors have attracted increasing attention [8]. A TCI (targeted covalent inhibitor) usually consists of two parts: a bond-forming functional group of low reactivity, which is commonly referred to as the “warhead,” and a selective noncovalent fragment for target recognition [8, 9]. The combination of covalent reaction and noncovalent interactions with the residues in the pocket for covalent inhibitors makes them possible to bind to many sites that are difficult to be targeted by noncovalent inhibitors alone [6]. One of the most representative examples is the discovery of covalent inhibitors for RAS (KRAS, NRAS, and HRAS), which is the most frequently mutated gene family in cancers and has been considered “undruggable” despite decades of extensive attempts to develop effective inhibitors [10–12].

The binding process of a TCI involves two steps. First, the noncovalent fragment selectively recognizes and binds to its target by favorable geometric and energetic complementarity. In the meantime, the warhead on the inhibitor is placed in an appropriate position relative to the nucleophilic residue around the pocket, which promotes the occurrence of the covalent-bond formation in the second step [13, 14]. Theoretically, the amino acids with nucleophilic groups in the side chains, such as cysteine [9, 15], serine [16, 17], lysine [18–20], and threonine [21], have the potential to react with covalent inhibitors. Among these amino acids, cysteine is the most popular one for TCI discovery owing to its intrinsic advantages, where the thiol group in cysteine can be deprotonated to thiolate with significantly increased nucleophilicity, making it the strongest nucleophile among the 20 canonical amino acids [22–24]. Besides, cysteine is usually noncatalytic and poorly conserved, which is beneficial for achieving high target selectivity [25], and the low-abundant nature of cysteine decreases the off-target risks of TCIs [26]. However, not every cysteine can be targeted by TCIs. Two necessary requirements need to be satisfied: (1) it should be close to a pocket to which an inhibitor can bind, and (2) the physicochemical property of the pocket environment is conducive to the thiol group deprotonation [26–28]. Weerapana and coworkers developed a quantitative proteomic method to profile the intrinsic reactivity of cysteine residues using a covalent probe, which labels cysteines with an electrophilic iodoacetamide group [29]. This study indicates that there is still a large number of cysteines in the proteome that could be utilized to design TCIs. The first step in structure-based covalent drug discovery is to find an effective covalent binding site, which, to some extent, defines the complicity and difficulty of the entire drug discovery process. Thus, it will be quite meaningful if we can resolve the paradigm of effective covalent binding sites from successful cases and predict the cysteine covalent ligandability using computational methods.

Over the past decade, deep learning (DL) has made unprecedented breakthroughs in tackling a broad spectrum of problems, such as protein structure prediction [30–33], protein function prediction [34, 35], drug virtual screening [36–42], and molecular generation [43, 44]. Though advances in biotechnology like high-throughput screening (HTS) and omics technology have provided a large amount of TCI data, DL methods have never been applied to the prediction of cysteine covalent ligandability. There are only a few computational studies on the factors affecting the cysteine acidity and reactivity [28, 45]. For example, Awoonor-Williams and Rowley calculated the p*K*a values of ligandable cysteines in kinases using thermodynamic integration based on molecular dynamics (MD) simulations [45], and they concluded that the acidities of ligandable cysteines within protein kinases are diverse and elevated, which are usually influenced by the degree of the solvation and electrostatic interactions with other charged residues. However, some studies pointed out that the accuracy of the methods in calculating the p*K*a of cysteine is similar to that of the null model, implying that these methods fail to accurately predict the reactivity of cysteines [46]. Huang et al. developed a GPU-accelerated continuous constant pH MD (CpHMD) method for more accurate and rapid prediction of protein p*K*a values based on independent pH [47, 48]. They applied this method to test the intrinsic reactivity of front pocket (FP) N-terminal cap (Ncap) cysteines in human kinases based on their p*K*a [28] and came to similar conclusions that hydrogen bonding and electrostatic interactions drive the reactivity, and their absence renders the Ncap cysteine unreactive. Soylu and Marino developed an energy- and knowledge-based method to predict cysteine reactivity using a decision tree model by evaluating the H-bond network and structure similarities [49]. Zhang et al. applied a support vector machine (SVM) to predict the covalent ligand-targeted cysteine residues [50], which was the first exploration to apply machine learning to cysteine ligandability prediction. A protein surface cavity detection method was used to find the pockets on protein surfaces, and the environmental features of cysteine residues were then extracted to develop a predictive SVM model, which achieved the performance with an accuracy of 0.73. However, the covalent ligandability of cysteines can be affected by many factors including the amino acid composition of the neighboring pocket, electrostatic characteristics of the cysteine environment, solvent exposure, and spatial orientation of the cysteine [27]. Predefined rules and/or descriptors that need extensive human expert knowledge were often used in traditional machine learning (ML) models, where the implied information from the original data may be missing [51]. DL exhibited strong capability in learning unique information from the primary data without human intervention [52, 53]. Recently, graph neural networks (GNNs) have drawn increasing attention and shown tremendous success in various application fields ranging from compounds toxicity prediction [54] to protein function prediction [55]. In GNN, atoms are treated as nodes and the relations between these atoms are represented by edges [56], which makes it possible to learn the complicated interactions among the atoms or groups from the original structures and to predict the covalent ligandability of cysteines.

Here, we proposed a novel deep graph learning framework, named Deep Covalent Site Identification (DeepCoSI), for detecting covalent-ligandable cysteines from the 3D structures of proteins, which significantly outperforms the method developed by Zhang et al. [50] The DeepCoSI model not only outlines the whole picture of the entire pocket but also focuses on the characteristics of cysteine itself. The predicted probability by DeepCoSI can reflect the influence of the key factors in a desired direction, implying that our model really learned the implicit paradigm of covalent-ligandable cysteines from the structures. Besides, two external test sets were constructed and utilized to validate the reliability of DeepCoSI in real application scenarios. Finally, DeepCoSI was applied to the entire set of protein structures in RCSB PDB to identify potential cysteines for covalent ligand discovery, and the database of the precomputed candidates was made publicly available to the scientific community.

## 2. Results

### 2.1. A Dataset for Benchmark

Due to the lack of a public benchmark for cysteine covalent ligandability prediction, we constructed a dataset for model development and evaluation. The dataset contains 1042 structures from the RCSB PDB belonging to 259 proteins. We detected 7490 cysteines on these protein structures, including 1076 cysteines bound with covalent ligands (positive samples) and 6414 flexible cysteines (negative samples). The number of the cocrystal structures for most proteins bound with covalent inhibitors is quite low (Supporting Information Figure S1a). However, multiple covalent inhibitors targeting a number of proteins from the peptidase C1 family [57], tyrosine-protein kinase family [58], coronaviruses polyprotein 1ab family [59, 60], and picornaviruses polyprotein family [61] have been reported, and relatively larger numbers of covalent-complex structures are available for these proteins (Supporting Information Figure S1c). The proportions of cysteines found in most protein chains are quite low (less than 5%), and the most frequent distribution interval appears in 0.025-0.03 (with the average of 0.028), indicating low abundance of cysteine among proteins (Supporting Information Figure S1b).

### 2.2. DeepCoSI to Outline the Pocket and Represent the Reactivity of Cysteines

The covalent ligandability of cysteine is determined primarily by the pocket environment and its intrinsic reactivity. And it is worth noting that the intrinsic reactivity of cysteine also depends on the surrounding environment which interacts with cysteine through H-bond, salt bridge, etc. [25, 48]. Therefore, it is of great importance to analyze and accurately encode the features of the pocket environment surrounding cysteines. Proteins are three-dimensional (3D) structures that consist of various atoms connected by covalent bonds and noncovalent interactions. The graph convolutional network (GCN) has been widely used in characterizing the structures of biomolecules, where the message from the neighboring nodes (atoms) can transmit to the central node (atom) through the edges (bonds or interactions) during the message passing stage, making it possible to capture the mutual effect between atoms [62–65].

To explore the framework of our model, we first built a preliminary GCN framework (PriDeepCoSI) to characterize the environmental features of the cysteine pocket (Supporting Information Figure S2). In PriDeepCoSI, the physicochemical and 3D information of the pockets were assigned to atoms and bonds, and the message processing stage allowed each atom to receive the information from its neighbors. The atom features were subsequently integrated into a vector to represent the properties of the entire pocket and used for predictions. In order to maximize the diversity between the datasets for model training and evaluation, we clustered the proteins based on their sequences with cd-hit [66] before splitting. Results showed that the performance of PriDeepCoSI was independent of the similarity between datasets (Supporting Information Figure S3), which would benefit to its application in real scenes, especially when the overlap of the spatial distributions between the predicted samples and the samples in the training set was insufficient. We further explored the influence of the pocket size on predictive accuracy and selected 15 Å for the subsequent study based on the AUPRC criteria (Supporting Information Figure S4) (details about PriDeepCoSI can be seen in Section 4.3).

**Figure 1:**
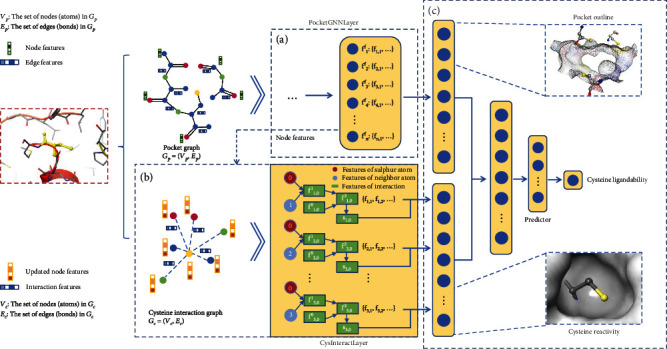
The workflow of DeepCoSI. (a) The PocketGNNLayer for message passing and atom state update which is the same as in PriDeepCoSI. (b) Another graph *G*_*c*_ is constructed to encode the noncovalent interaction between the thiol group and other atoms in pockets. *V*_*c*_ and *E*_*c*_ denote the set of nodes (atoms) and edges (bonds) in *G*_*c*_, respectively. CysInteractLayer accepts the final node features from PocketGNNLayer and aggregates the interaction information. (c) The readout from PocketGNNLayer to represent pocket outline and the readout from CysInteractLayer to represent cysteine reactivity are combined to predict the cysteine ligandability (the ability of the cysteine to be targeted by a covalent ligand, which was represented by a probability value output by model).

The readout operation of PriDeepCoSI outlined the profile of the entire pocket but failed to capture the characteristics of cysteine itself. The reactivity of cysteine is an essential factor for accurate prediction of ligandability and is primarily determined by the noncovalent interaction with the surrounding environment [29, 45, 49, 67]. Therefore, on the basis of PriDeepCoSI, we constructed DeepCoSI (Figure 1) by adding another graph to describe the interaction between the thiol group of cysteine and the surrounding environment. The interacting atom was defined based on the distance between the sulphur atom of cysteine and the atom in the pocket, and the specific form of interaction was learned by the model itself. The interaction vectors were calculated by the cysteine-interaction graph based on the atom features generated from PocketGNNLayer (see Section 4.4 for details). All the interactions with the thiol groups were assembled into a vector to characterize the reactivity of cysteine. Finally, the covalent ligandability of cysteine was predicted based on the information of both the pocket environment and the reactivity of cysteine.

**Figure 2:**
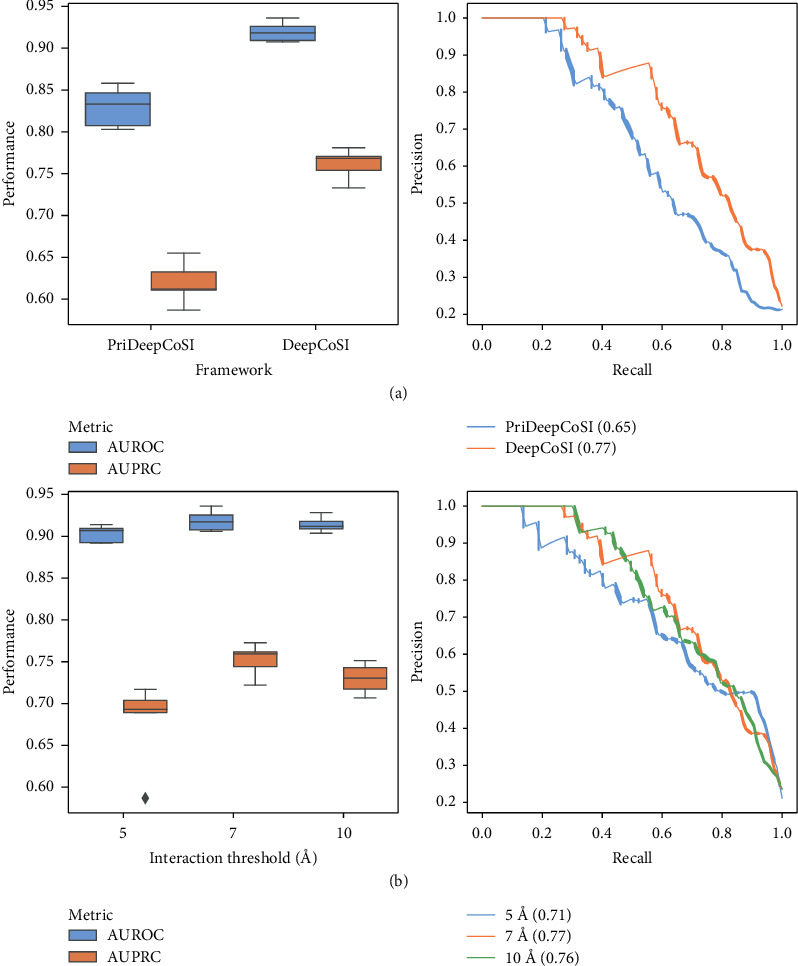
(a) The performance comparison between DeepCoSI and PriDeepCoSI. (b) The performance of DeepCoSI with different interaction thresholds.

The performance of the two frameworks was directly compared, and the results are shown in Figure 2(a) and Supporting Information Table S3. In both evaluation metrics, DeepCoSI significantly outperformed PriDeepDoSI. The AUROC values from DeepCoSI and PriDeepDoSI were 0.83 and 0.92, respectively, which indicated that introducing the interaction network of the thiol group to the framework was successful and improved the accuracy of predictions.

We further explored the influence of the defined interaction distance (5 Å, 7 Å, and 10 Å) on the performance of the model (Figure 2(b) and Supporting Information Table S4). The AUROC and AUPRC values were found to be the lowest when the threshold distance was set to 5 Å. The model with the threshold values increased to 7 Å exhibited higher predictive accuracy (AUROC = 0.92, AUPRC = 0.76). However, increasing the threshold distance to 10 Å failed to improve the accuracy, implying that the interactions beyond 7 Å were too weak to have substantive impact on this task.

### 2.3. DeepCoSI versus Feature-Based Traditional Model

**Figure 3:**
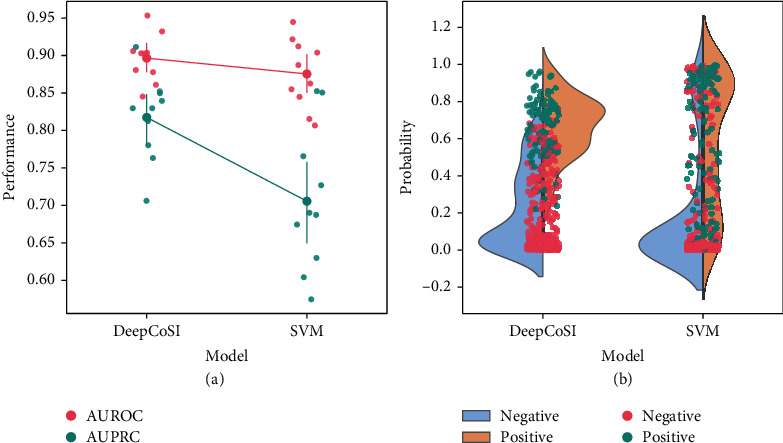
(a) The performance comparison between DeepCoSI and SVM model. (b) The distribution of the predicted probabilities by the DeepDoSI and SVM models.

Zhang et al. [50] established and reported a classification model by SVM, which was the only machine learning (ML) model to predict the ligandability of cysteine. They calculated and manually selected some features to characterize the properties of cysteine and the surrounding environment. We built a similar SVM model and DeepCoSI using the same dataset and compared the predictive performance of the two models (see Section 4.6 for details).  Figure 3 and Supporting Information Table S5 show the results from 10 independent running. The average AUPRC values for DeepDoSI and the SVM model were 0.82 and 0.71, respectively, indicating that the predictive accuracy of DeepDoSI was significantly higher than that of the SVM model. We further analyzed the distribution of the probability values of both the positive and negative samples. For the SVM model, the predicted values of most negative samples were distributed from 0 to 0.2, but the probability values of the positive samples were evenly scattered throughout 0 to 1, indicating the SVM model failed to identify ligandable cysteines. For DeepCoSI, the distributions of the positive samples (0.5-0.8) and negative samples (0-0.4) were significantly different. DeepCoSI exhibited enhanced ability in predicting the ligandable cysteines from protein structures compared with the feature-based SVM model.

### 2.4. Can DeepCoSI Learn Hidden Paradigm of Covalent-Ligandable Cysteines?

DL is an incomprehensible black box, which makes it difficult for us to figure out what happens inside the box [68, 69]. One way to test whether the model has learned the hidden paradigm of covalent-ligandable cysteines is to modify the input in a specific direction to investigate its ability to accurately reflect the influence of some known task-related factors on the prediction results. There are some factors that can affect the binding of cysteine to covalent inhibitors, including the electrostatic interactions [45] and spatial orientation of the thiol group [70].

**Figure 4:**
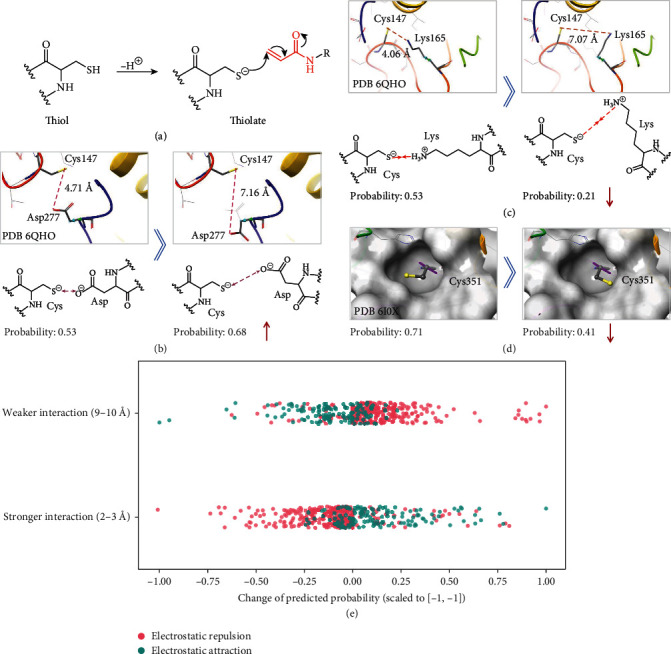
Changes in the predicted value after structure modification. (a) The deprotonation of cysteines before covalent linking with ligands. (b) Structure modification on PDB 6QHO to decrease the electrostatic repulsion between Cys147 and Asp277. (c) Structure modification on PDB 6QHO to decrease the electrostatic attraction between Cys147 and Lys165. (d) Structure modification on PDB 6I0X to change the orientation of cysteine from towards the pocket cavity to towards the pocket edge. (e) Statistics study on model's response to the changes in electrostatic interaction. The distance between charge centers represents the strength of interactions.

Before reacting with covalent inhibitors, the thiol group of cysteine is deprotonated to form a thiolate (Figure 4(a)). The electrostatic interaction affects the stability of thiolate that determines the probability of covalent linking. The existence of the negative charges in the environment brings about the electrostatic repulsion and reduces the stability of thiolate, while positive charges can form stable salt bridges with thiolate that increase the concentration of the ionic form in the conversion equilibrium [45]. Therefore, we first explored whether the model was sensitive to changes of the electrostatic interactions. We randomly selected three samples from the test set, in which the cysteine group was in close contact with the negatively charged aspartic acid. The dihedral angle and the distance between charge centers were then modified to change the strength of the electrostatic interaction. For PDB 6QHO, we adjusted the dihedral angle of Asp277 from -116.7° to 73.3° with the distance between the charge centers changing from 4.71 Å to 7.16 Å. The reduction of the repulsion effect led to increased predicted probability from 0.53 to 0.68 (Figure 4(b)). Similarly, for PDB 6I0X, the dihedral angle of Asp130 was adjusted from -63.4° to 134.6° and the distance between the charge centers increased from 6.05 Å to 9.16 Å, improving the probability from 0.71 to 0.85 (Supporting Information Figure S5a). The dihedral angle of another negative charge center in the pocket, Asp347, was rotated from 176.4° to 26.4°, and the model gave a higher prediction value (from 0.71 to 0.84) (Supporting Information Figure S5b). Similar results were obtained by modifying the structure of 4QBB (Supporting Information Figure S5c). The results indicated that reduction of the electrostatic repulsion between the thiolate and the surrounding environment could improve the predicted probability. Two positively charged amino acids (Lys165 and Arg178) near Cys147 could form stable salt bridges with thiolate. Changing the dihedral angle of Lys165 from -170.4° to -86.4° significantly decreased the predicted probability (from 0.53 to 0.21) (Figure 4(c)). As for Arg178, the predicted value slightly decreased from 0.53 to 0.41 after structural change (Supporting Information Figure S5d). This demonstrated that our model was sensitive to the change of salt bridge which might affect the prediction accuracy. Another factor that affects the binding of covalent ligands is the spatial orientation of cysteine [70]. The orientation of Cys351 in the structure of 6I0X was reversed by pointing to the pocket edge. This adjustment was sterically unfavorable for the binding of covalent inhibitors, and the predicted value of the model decreased from 0.71 to 0.41 (Figure 4(d)). Likewise, rotating the dihedral angle of Cys51 in the structure of 4QBB from 70.7° to 97.9° decreased the predicted probability (from 0.79 to 0.66) (Supporting Information Figure S5e). The results of five independent repeated runs can be seen in Supporting Information Table S6.

In addition to the case study, we further statistically analyzed the response of our model to changes in knowledge-based factors related to the task. We randomly adjusted the distance between cysteine and its surrounding charge centers to modify the strength of the electrostatic interactions (see Section 4.7 for details). As we expected, the changes on different types of interactions could have opposite effects on the prediction results (Figure 4(e)). Our model tended to give higher probability to the structures with weaker electrostatic repulsion which could cause the instability of thiolate. On the contrary, the salt bridge between thiolate and positive charge center could stabilize the ionic form of cysteine and this preference could also be reflected by our model. The above results showed that our model could capture the impact of the task-related factors without the input of any defined information in the training process, which on the other hand indicated that the hidden paradigm of covalent-ligandable cysteines was learned by the model.

### 2.5. How Does DeepCoSI Perform in Real Application Scenarios?

**Figure 5:**
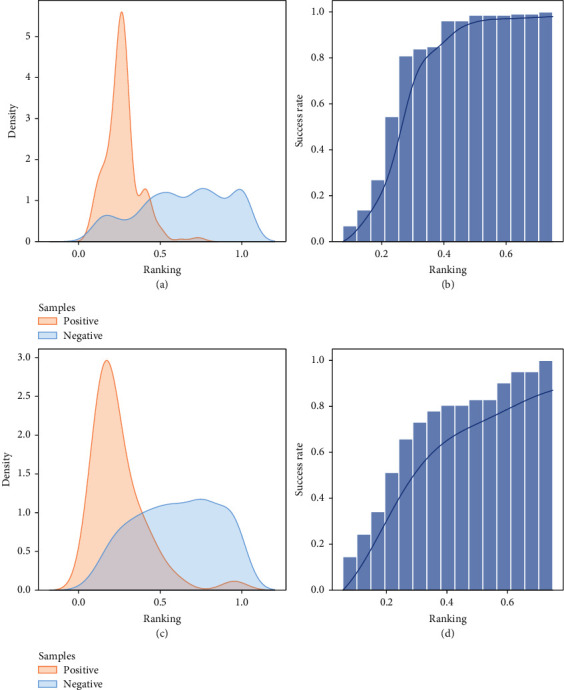
The performance of DeepCoSI in real application scenarios. (a) External test set 1: the distribution of the normalized ranking according to the probability predicted by DeepCoSI. (b) External test set 1: the cumulative curve of the success rate when setting different criteria. (c) External test set 2: the distribution of the normalized ranking according to the probability predicted by DeepCoSI. (d) External test set 2: the cumulative curve of the success rate when setting different criteria.

In real application scenarios, it is critical to know which cysteine should be selected to design covalent inhibitors. An efficient model should be able to accurately identify the ligandable cysteines from protein structures. In order to test the predictive ability of the model, we constructed another external test set (see Section 4.8 for details), in which the covalent ligands were not contained in the protein structures (external test set 1). We ranked the cysteines in each structure based on the probability given by the model (Supporting Information Table S7). The rankings were normalized according to the total number of samples in each structure.  Figure 5(a) shows the ranking distribution of the positive and negative samples. The rankings of the positive samples were mainly distributed around 0.25, while the negative samples were scattered in the interval of 0.5-1. This demonstrated that our model could effectively distinguish the ligandable cysteines from nonligandable cysteines. We further explored the success rates of prediction by setting different threshold values (Figure 5(b)). When the threshold was set to 0.25, the success rate was 54%, and it quickly increased to 81% when the threshold was set to 0.3. The ligandable cysteines in 98% of the structures could be identified when the threshold was set to 0.5. The results showed that our model could efficiently identify ligandable cysteines from the apo structures of proteins, which provided guidance to covalent site selection in real application scenarios.

We further validated the prediction ability of our model with chemical proteomics data. Backus et al. used competitive isoTOP-ABPP to probe the ligandability of cysteines in the human proteome and identified 758 liganded cysteines on 637 distinct proteins [67]. We searched their structures with UniProt ID in RCSB PDB, and 41 structures that satisfied the filtering criteria (see Section 4.8 for details) were collected (external test set 2). Likewise, we used our model to rank the cysteines in each structure in order to evaluate its ability to identify ligandable cysteines (Figures 5(c) and 5(d), Supporting Information Table S8). The prediction performance on this dataset slightly decreased but was still acceptable. The ranking distribution of the positive and negative samples focused on diverse region. The success rate was 51.2% when the threshold was set to 0.25, and it would increase to 82.9% when the threshold was set to 0.5. The ligandable cysteines in 21 structures (51.2%) could get the highest predicted probability, and it would go up to 31 (75.6%) when considering the top two predictions. This result showed that DeepCoSI could be used as an alternative approach to probe the ligandability of cysteines *in silico*, especially for researchers who cannot afford the competitive isoTOP-ABPP.

### 2.6. Mapping the Ligandability of Cysteines in the Entire Database of PDB

So far, the RCSB PDB [71] has collected more than 180,000 structures of biological macromolecules, and it provides a wealth of information for biological and pharmaceutical studies. It would be quite important to make full use of the structural data for developing novel covalent inhibitors. Thus, DeepCoSI was applied to predict the ligandability of cysteines in the entire PDB database. 40,098 structures with the resolution of less than 2 Å and 144,938 cysteines without disulfide bond or ligand binding were finally selected (see Section 4.9 for details). 33% of the structures are of human proteins, and the rest span many other organisms, including rodents, bacteria, and viruses. We ranked these cysteines in each structure according to the predicted probability and uploaded these profiled data to CovalentInDB [72] (http://cadd.zju.edu.cn/cidb/deepcosi/cys), which is a comprehensive covalent inhibitor database for public use.

We further validated the reliability of the profiled database with the evidence from existing biological experiments. In addition to analyzing the crystal structures, other methods, such as mass spectrometry and point mutation, can also be used to verify the binding of covalent ligands. We collected the unbound structures of the proteins that were experimentally validated to be able to bind with covalent inhibitors. In order to evaluate the ability of the model to distinguish ligandable cysteines from the others, 11 proteins that contain more than 3 cysteines were included in our profiled data. The prediction results showed that the ligandable cysteines in 8 structures could get the highest predicted probability, which achieved a high success rate of 72.7% (Table 1). We also noted that DeepCoSI was sensitive to the input structures. The Cys1045 residue in VEGFR-2 [78] could be successfully identified by the structure of 2P2H (ranked 1/8) but was ranked 3/8 by using the structure 3WZE. Further analysis showed that the direction of Cys1045 was different in these two structures. The former cysteine pointed to the outside of the pocket, which was beneficial to the binding of covalent inhibitors, while the latter pointed to the inside of the pocket (Supporting Information Figure S6). This suggested that the use of multiple conformations might improve the accuracy of predictions, which could be considered in future investigations.

## 3. Discussion

Due to some intrinsic advantages, including long residence time, high selectivity, and strong binding affinity, covalent ligands are attracting more and more attention in drug discovery [72, 84]. However, a lack of the knowledge of covalent binding sites has limited the development of covalent ligands. At present, studies of covalent inhibitors are largely restricted to some specific protein classes, including kinases, proteases, and beta-lactamases [72]. Therefore, identifying potential covalent binding sites within the proteome will greatly expand the scope of covalent ligand research. The isoTOP-ABPP (isotopic tandem orthogonal proteolysis–activity-based protein profiling) provides a strategy to quantitatively map the intrinsic reactivity of cysteine and lysine from an experimental point of view [19, 29]. However, the profile results are closely related to the structures of the probes, implying that larger compound libraries are needed to achieve more comprehensive screening. A ligand-free method should be able to discover more general paradigms of ligandable residues, thereby expanding the scope of screening targets and covalent sites. Here, we describe a DL method, DeepCoSI, that uses protein structural data to predict the ligandability of cysteine. Based on the physicochemical and 3D information extracted from the protein structures, our model was able to characterize both the overall environment of the cysteine pocket and the reactivity of cysteine. The structural modification experiment further revealed that DeepCoSI was sensitive to changes of the key factors related to cysteine ligandability in a desired direction. This also indicated the strong feature extraction ability of DL, which was not realized by feature-based methods. The test on real application scenarios demonstrated that our model could effectively identify ligandable cysteines from protein structures. Mapping of the ligandability of cysteines based on the entire database of PDB provided valuable clues for further design and discovery of covalent inhibitors.

DeepCoSI was developed and committed to predict the ligandability of cysteines in protein structures. However, the binding of covalent inhibitors largely depends on the noncovalent interaction and geometric complementarity between protein and ligand [8, 29, 67, 85]. Therefore, it is important to include both the ligandability of cysteines and the nonbonded interactions of protein/ligand complexes in assessing the activity of covalent inhibitors. Besides, although DeepCoSI can effectively characterize the contacts between atoms in the pocket, the protein structures processed by our model are static, which may not reflect the actual state of the proteins in the biological system [25, 48]. Considering protein flexibility in the model may help improve the predictive accuracy by combining DeepCoSI with sampling methods, e.g., Monte Carlo or MD simulation, where different conformations of protein structures can be generated. In addition to cysteine, some other nucleophilic amino acids can also be used to develop covalent ligands, including serine [16], lysine [18, 19], and threonine [21]. However, the number of reported covalent inhibitors that are designed based on these residues is quite limited, making it difficult to develop reliable predictive models. Transfer learning techniques enable the application of DeepCoSI into other nucleophilic residues with high abundance, which will provide more options and opportunities for developing novel covalent ligands.

In conclusion, we describe a method to identify ligandable cysteines from protein structures, which is a primary problem that restricts the design and development of covalent ligands. The ligand-free DeepCoSI identifies a large number of potential covalent binding sites based on the structures from the entire PDB database and provides new insights for studying protein functions and designing novel covalent drugs.

## 4. Methods

### 4.1. Construction of Benchmark Dataset

We collected the cocrystal structures bound with covalent ligands from the RCSB PDB [71]. In order to ensure the integrity of the dataset, we downloaded the whole database and identified all cysteines that form covalent bonds with ligands using in-house scripts. These cysteines were regarded as the positive samples while other flexible cysteines in the same chain were regarded as the negative samples. Subsequently, we used UCSF Chimera [86] to extract all amino acids within a certain distance from each cysteine as the surrounding environment (defined as “the pocket of cysteine”), which would be used as the input of our model.

### 4.2. Splitting of the Dataset

To avoid aggregation of samples with high similarity in the training set, validation set, or test set, we used cd-hit [66] to cluster proteins according to their sequences (Supporting Information Figure S3). We collected the sequence of each protein from the UniProt [87]. We controlled the strictness of clustering by setting different values of identity (40%, 60%, and 80%). According to the recommendation of cd-hit, we used different word sizes for different thresholds during clustering (*n* = 2 for threshold 40%, *n* = 4 for threshold 60%, and *n* = 5 for threshold 80%). Other parameters were set to default. After clustering, the dataset was randomly split (training set : validation set : test set = 8 : 1 : 1), and the proteins from the same cluster could only appear in one of the datasets.

### 4.3. The Workflow of the Preliminary Model (PriDeepCoSI)

PriDeepCoSI consisted of three main components: (1) graph generation and embedding with physicochemical and 3D information, (2) message passing and hidden state update via PocketGNNLayer (to update the properties of central atoms based on the influence of surrounding atoms), and (3) graph pooling (to aggregate the information from all atoms into a vector) and final classification via a fully connected layer.

In the first step, amino acids within a certain distance (10 Å, 15 Å, or 20 Å) from cysteine were set as the environment (pocket). Then, the environment was transformed into an atom-level pocket graph (*G*_*p*_ = (*V*_*p*_, *E*_*p*_)). The corresponding adjacency matric, *A*_*i*,*j*_^*p*^ ∈ ℝ^*L*×*L*^, was defined as follows:

(1) $$\begin{array}{c} A_{ij}^{p}=\left\{\begin{array}{c} 1,\text{if atom }i\text{ and }j\text{ are covalent}-\text{bonded},\\ 0,\text{otherwise}, \end{array}\right. \end{array}$$

where *L* is the number of the heavy atoms in this pocket. In order to characterize the physicochemical properties and 3D structural characteristics of the pocket with a graph, we embed the nodes and edges with the corresponding features, respectively (Supporting Information Table S9). The initial node features consisted of two parts: 2D features with atomic physicochemical properties calculated by RDKit [88] and 3D features to reflect the surrounding environment of each atom. The 3D features were calculated by the symmetry functions proposed by Smith et al. [89–91], which could represent the local chemical environment accounting for both radial and angular features. These features only depend on the distance between any two atoms and the angle formed by any three atoms in the pocket. Similarly, the initial edge feature was also composed of two parts: 2D features with bond properties calculated by RDKit and 3D features including bond length and bond positions [92].

In the second step, the PocketGNNLayer was used to pass a message through bonds and to get the final state of atoms. We adopted the attention mechanism (to assign different weights to neighbor atoms when their message is transferred to the central atom) proposed by Attentive FP [56] to reflect the difference in the impact of neighbor atoms on the central atom. PocketGNNLayer consisted of three GCN layers, where aggregation of neighboring information and update of atom hidden state were accomplished. The calculation process in *l*^th^ layer is as follows:

(2) $$\begin{array}{c} u_{ij}^{l}=\text{LeakyReLU}\left(w_{1}^{l}\left[f_{i}^{l-1}\left\|f_{j}^{l-1}\right.\right]\right),\;w_{1}^{l}\in {\mathbb{R}}^{1\times 2D}, \end{array}$$

(3) $$\begin{array}{c} s_{ij}^{l}=\frac{\exp\left(u_{ij}^{l}\right)}{\sum_{k\epsilon N_{\left(i\right)}}\exp\left(\;u_{ik}^{l}\right)}, \end{array}$$

(4) $$\begin{array}{c} f_{i}^{l}=\text{BN }\left(\text{ReLU}\left(\text{GRU}\left(\text{ELU}\left(\underset{k\epsilon N_{\left(i\right)}}{\sum }s_{ik}^{l}w_{2}^{l}f_{k}^{l-1}\right),f_{i}^{l-1}\right)\right)\right),\;w_{2}^{l}\in {\mathbb{R}}^{D^{\prime }\times D}, \end{array}$$

(5) $$\begin{array}{c} f_{i}^{f}=\overset{L}{\underset{t=1}{\sum }}f_{i}^{t}. \end{array}$$

The message from neighbors was transferred to atom *i* in a weighted way calculated by the attention mechanism as shown in Equations (2) to (4). In Equation (2), *u*_*ij*_^*l*^ is an unnormalized attention score determined by the hidden state of nodes *i* and *j* in (*l* − 1)^th^ layer and *D* is the length of the hidden state in (*l* − 1)^th^ layer. *s*_*ij*_^*l*^ in Equation (3) denotes the normalized attention score calculated by the softmax function, where *N*_(*i*)_ is the collection of neighbor nodes of node *i*. Equation (4) was used to aggregate the information from *N*_(*i*)_ with the attention score *s*_*ij*_^*l*^ and updated the hidden state of atom *i* by fusing the incoming message and previously hidden state *f*_*i*_^*l*−1^ with GRU. *D*′ denotes the length of the hidden state in the *l*^th^ layer. Instead of using the hidden features from the last GCN layer, the final node representation for atom *i*, *f*_*i*_^*f*^, was calculated by aggregating the node hidden features in each layer as described in Equation (5), where *L* is the number of GCN layers and *f*_*i*_^*t*^ denotes the hidden stats in the *t*^th^ layer. This equation was used to prevent the oversmooth issue where the representations of nodes tend to be more similar with the increasing number of GCN layers.

In the third step, the final pocket representation, *f*_*p*_, was obtained by performing a global pooling layer (to aggregate the information from all atoms into a vector and outline the profile of the entire pocket) as shown in the following:

(6) $$\begin{array}{c} f_{p}=\overset{N}{\underset{i}{\sum }}w_{3}f_{i}^{f}\cdot f_{i}^{f},\;w_{3}\in {\mathbb{R}}^{1\times D^{f}}, \end{array}$$

where *w*_3_*f*_*i*_^*f*^ is the importance weight of atom *i* calculated from *f*_*i*_^*f*^, *N* is the number of atoms in the pocket, and *D*^*f*^ denotes the vector length of *f*_*i*_^*f*^. Then, a fully connected layer with a LeakyReLU activation function was used to compute the hidden representation from the pooled representation and output the probability (pocket ligandability: the ability of the pocket to accommodate a ligand) with the sigmoid function:

(7) $$\begin{array}{c} \text{probability }\left({\hat{y}}_{i}\right)=\text{sigmoid}\left(\text{MLP }\left(f_{p}\right)\right). \end{array}$$

### 4.4. The Workflow of the DeepCoSI

The reactivity of cysteine is a critical factor affecting its covalent ligandability. In order to reflect the interactions between the cysteine and the surrounding environment, we developed DeepCoSI based on the preliminary model. Compared with the preliminary model, two changes were introduced to DeepCoSI.

1. Another graph (*G*_*c*_ = (*V*_*c*_, *E*_*c*_)) was constructed to represent the noncovalent interaction between the thiol group of cysteine and the surrounding atoms in the pocket. The corresponding adjacency matric, *A*_*i*,*j*_^*c*^ ∈ ℝ^*L*×*L*^, was defined as follows:

(8) $$\begin{array}{c} A_{i,j}^{c}=\left\{\begin{array}{c} 1,\text{if atom }i\;{\text{is}}^{\text{“}}{\text{S}}^{\text{”}}\text{ of cysteine and }d_{ij}<7\;\overset{{}^{\circ}}{\text{A}},\\ 0,\text{otherwise}, \end{array}\right. \end{array}$$

where *d*_*ij*_is the distance between atoms *i* and *j*.

(2) A CysInteractLayer was added to encode and aggregate the interaction information:

(9) $$\begin{array}{c} f_{ij}^{2}=\text{MLP }\left(\left[f_{ij}^{0}\left|\;\right|\left(f_{i}^{f}+f_{j}^{f}\right)\right]\right), \end{array}$$

(10) $$\begin{array}{c} s_{ij}=\text{Tanh}\left(w_{4}f_{ij}^{2}\right),\;w_{4}\in {\mathbb{R}}^{1\times D^{2}}, \end{array}$$

(11) $$\begin{array}{c} f_{c}=\overset{N}{\underset{i,j}{\sum }}s_{ij}f_{i,j}^{2}. \end{array}$$

Equation (9) was used to encode the interaction information between atoms *i* and *j*. *f*_*i*_^*f*^ and *f*_*j*_^*f*^ are the final features of atoms *i* and *j* passed from PocketGNNLayer; *f*_*ij*_^0^ denotes the initial feature of edge, and *D*^2^ denotes the vector length of *f*_*ij*_^2^, which is the final characterization of the interaction between atoms *i* and *j*. Finally, all the interactions with the atom “S” were aggregated by the same pooling method that was used in PriDeepCoSI (Equations (10) and (11)). The vectors *f*_*p*_ obtained from PocketGNNLayer and *f*_*c*_ obtained from CysInteractLayer represent the outline of the whole pocket and the reactivity of cysteine (especially the thiol group on the side chain that forms the bond with the covalent ligand), respectively.

(12) $$\begin{array}{c} f_{t}=\text{Tanh}\left(w_{5}f_{p}\right)f_{p}+\text{Tanh}\left(w_{5}f_{c}\right)f_{c},\;\;w_{5}\in {\mathbb{R}}^{1\times D^{2}}, \end{array}$$

(13) $$\begin{array}{c} \text{probability }\left({\hat{y}}_{i}\right)=\text{sigmoid}\left(\text{MLP }\left(f_{t}\right)\right). \end{array}$$

Then, the two types of information were combined in a weighted way (Equation (12)) and the final prediction of the cysteine ligandability (the ability of the cysteine to be targeted by a covalent ligand, which was represented by a probability value) was carried out by a fully connected layer and the sigmoid function (Equation (13)).

### 4.5. Model Training and Evaluation

Our model was implemented by the open-source DGL-CUDA11.1 (Version: 0.7.1) [93] with PyTorch (Version: 1.8.0+cuda11.1) as the backend and RDKit (Version: 2018.09.3) [88] python packages. To account for imbalanced labels, both PriDeepCoSI and DeepCoSI were trained to minimize the weighted binary cross-entropy cost function (focal loss) that gives higher weights to the class with fewer training examples:

(14) $$\begin{array}{c} L\left(\Theta \right)=-\frac{1}{N}\overset{N}{\underset{i=1}{\sum }}\alpha {\left(1-{\overset{\wedge }{y}}_{i}\right)}^{\gamma }\log{\hat{y}}_{i}, \end{array}$$

where Θ is the set of all parameters in all layers to be learned; *N* is the total number of the samples in the dataset; ${\hat{y}}_{i}$ is the predicted probability for sample *i*; *α* is the weighting factor in balancing the importance of positive and negative samples and was set as No. of negative samples/No. of all samples; *γ* is the focusing parameter used to adjust the rate of downweighted easy-classified samples and was set to 2.0 in our experiment. To avoid overfitting, an early stopping criterion was used with patience = 70 (i.e., the training process would be terminated if the validation AUROC does not improve in 70 epochs). A learning rate (lr) of 0.0003 and a batch size of 8 were used in the ADAM optimizer, and the default number of epochs was set to 1000.

The performance of models was evaluated by the area under the receiver operating characteristics curve (AUROC) and the area under the precision-recall curve (AUPRC). Since our positive and negative samples were unbalanced, AUPRC was used as the main metric for evaluation since it is sensitive to changes in class distribution.

### 4.6. Comparison with Feature-Based Traditional Method

Here, we used the method proposed by Zhang et al. to generate the features and then developed the SVM model [50]. Two types of features (physicochemical descriptors and Tanaka descriptors) were used to characterize cysteine and its environment in Zhang et al.'s study (Supporting Information Table S10). First, we detected the pockets around the protein with CAVITY (1.1) [94], a protein surface cavity detection and druggability analysis program. If the cysteine was within a CAVITY detected pocket, the property of the pocket, including p*K*_dAve_, hDVR, hbVR, and lipVR, would be calculated by CAVITY. Then, we used in-house scripts to count the number of each type of amino acid within a certain distance from the cysteine. The 20 amino acids were divided into 13 categories according to Tanaka alphabet, which was originally used for protein design. We also calculated the SASA (solvent accessible surface area) and p*K*a of cysteine as the features using FreeSASA (2.1.0) [95] python packages and PROPKA3 [96], respectively. It is worth noting that if a cysteine existed in multiple pockets at the same time, we would select the pocket with the largest p*K*_d_ value for feature calculation.

The dataset used for comparison was smaller than the benchmark we built because some cysteines failed to pass the feature calculation stage (pocket detection by CAVITY and p*K*a calculation). We randomly split the dataset 10 times using the methods described in Section 4.2. The training method for DeepCoSI was the same as Section 4.5. As for SVM, we chose the commonly used radial basis function (RBF) and optimized the hyper parameters C (0.01 to 1) and gamma values (0.0001 to 0.01) using the Bayesian optimization. The parameters with the highest performance on the validation set were chosen for the final model.

### 4.7. Structure Modification Experiment

We directionally modified the pocket structures of cysteines to study whether our model has learned the hidden paradigm of covalent-ligandable cysteines. For the case study, we used Schrödinger (Version 2019) to adjust the dihedral angle of amino acids to change the strength of the interaction and the spatial orientation of cysteines. For statistics study, we adjusted the strength of the electrostatic interaction by changing the distance between the electrostatic centers, which can be represented by the edge feature in the cysteine noncovalent interaction graph. We regarded the oxygen anion on the carboxyl group of glutamic acids and aspartic acids as a negative charge center and nitrogen anion of lysines and arginines as a positive charge center. To simulate a weaker interaction, we randomly set the distance between 9 and 10 Å. To simulate a stronger interaction, it was set to 2-3 A.

### 4.8. Construction of External Test Datasets

We built two external test sets to assess the predictive ability of our model in actual application scenarios. External test set 1: after splitting the baseline (Section 4.5), we researched the crystal structures of proteins in the test set in the RCSB PDB. Unlike the baseline, no covalent ligands were included in these crystal structures, and the positive samples were in a flexible state, which was consistent with the actual application scenario. A resolution threshold of 2.5 Å for these crystal structures was applied, and the cysteine pockets were then extracted and used for the subsequent predictions. Please refer to SI for more details of this dataset. External test set 2: as for the chemical proteomics data, we searched the RCSB PDB with UniProt IDs which were provided by the original literature [67]. To ensure the quality of structures, we filtered only the protein structures that have a resolution below 2 Å. For those proteins with more than one PDB entry, the most complete one structure (covers the most amino acids) was preserved. Those structures in which the ligandable cysteine cannot be found at the corresponding position that was mentioned in the literature were excluded. To evaluate the ranking power of our model, only structures with more than 3 cysteines were preserved for success rate analysis.

### 4.9. Prediction on Structures from the PDB

To ensure the quality of structures, we filtered only the protein structures that have a resolution below 2 Å. We removed all atoms except those from amino acids. Then, we extracted the information of cysteines from each structure. We only kept cysteines with a free thiol group and removed all that formed disulfides or covalently attached to a ligand. We further removed cysteines which had more than one copy per chain in the structure to prevent redundancy. Finally, predictions were carried out by using DeepCoSI.

## Figures and Tables

**Table 1:** The result from the profiled database by DeepCoSI.

Protein	PDB	Cys	Ranking	Num_Cys^a^	Reference
O43318	7NTH	A-174	1	9	Ref. [73]
P14900	2Y67	A-413	1	5	Ref. [74]
P16455	1QNT	A-145	1	4	Ref. [75]
P20582	3H76	A-112	1	5	Ref. [76]
P29350	4HJP	A-453	1	5	Ref. [77]
P35968	2P2H	A-1045	1	8	Ref. [78]
P61077	1X23	A-85	1	4	Ref. [79]
Q9BY41	5THV	A-153	1	9	Ref. [80]
P10828	6KKB	X-309	3	7	Ref. [81]
P41182	6TOK	A-53	2	5	Ref. [82]
Q15118	2Q8G	A-240	4	4	Ref. [83]

^a^Total number of the flexible cysteines in structure.

## Data Availability

The dataset and source code are available at -https://github.com/Brian-hongyan/DeepCoSI. The profiled data are available at - http://cadd.zju.edu.cn/cidb/deepcosi/cys.

## Supplementary Materials

Supplementary 1 Supporting Information 1 (Word): description of details of PriDeepCoSI, multiple independent repeated runs, and validation on external datasets. Includes Supporting Information Figures (S1-S6) and Supporting Information Tables (S1-S10) cited inside the manuscript.

Supplementary 2 Supporting Information 2 (XLSX): includes details of the dataset for benchmark (Sheet 1) and the clustering result from cd-hit (Sheet 2).
