# Supplementary material for: Proteome-Wide Profiling of the Covalent-Druggable Cysteines with a Structure-Based Deep Graph Learning Network
Source: Research (Wash D C). 2022 Jul 21;2022:9873564. doi: 10.34133/2022/9873564 (PMC9343084; doi:10.34133/2022/9873564)
Supplement: Supplementary 1 — Supporting Information 1 (Word): description of details of PriDeepCoSI, multiple independent repeated runs, and validation on external datasets. Includes Supporting Information Figures (S1-S6) and Supporting Information Tables (S1-S10) cited inside the manuscript. [file 9873564.f1.docx]

**Supporting information**


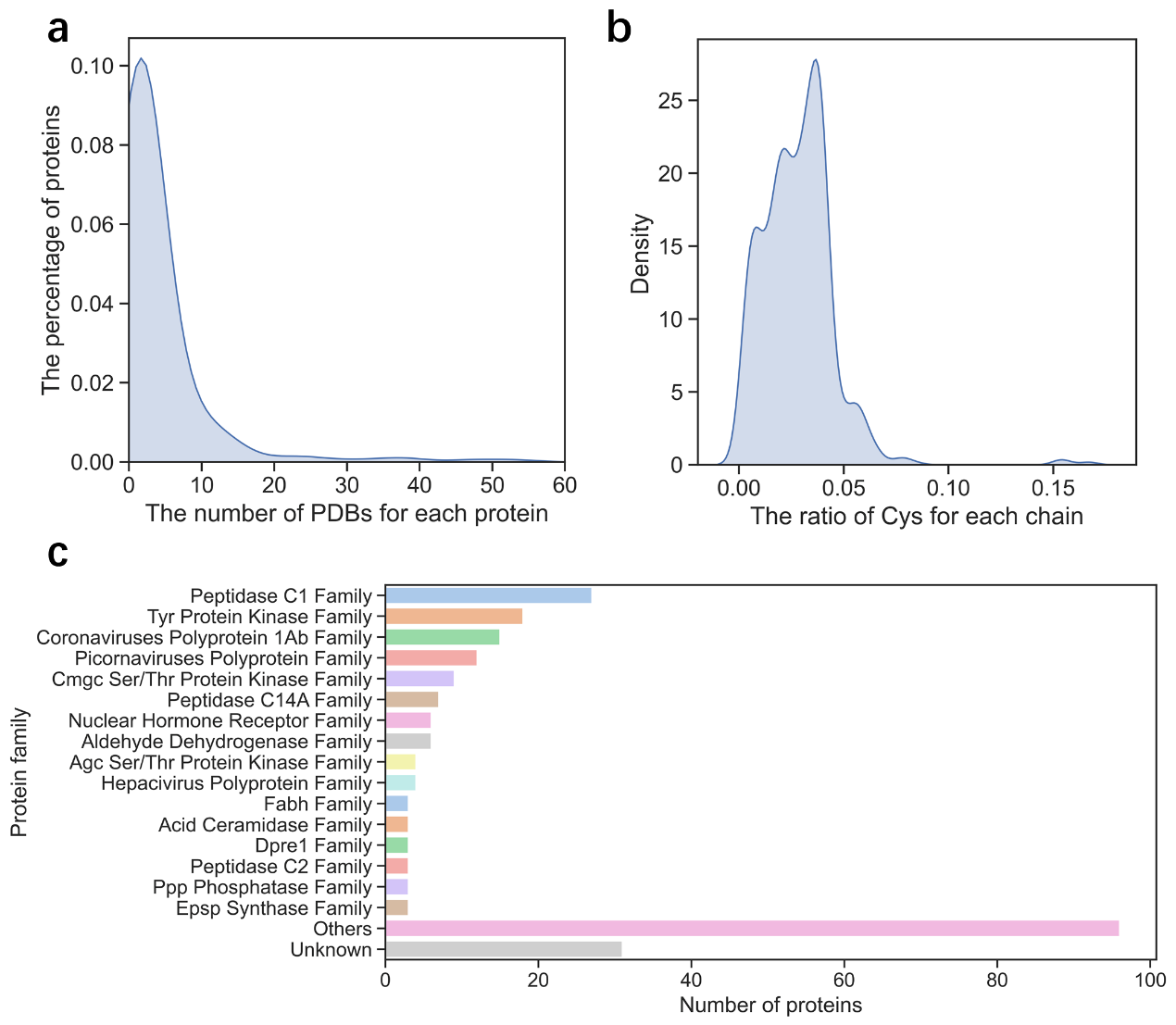


**Figure S1.** Data statistics on the benchmark. (a) The distribution of the number of PDBs for each protein in the benchmark. The SARS-CoV-2 3CL protease is excluded from the statistics, which have far more PDBs (119) than other proteins. (b) The distribution of the ratio of cysteines in each PDB chain. For protein structure with multiple chains, only the chain linked with covalent inhibitor is considered. (c) The classification of proteins based on family annotation in UniProt. This is the initial data collected from the PDB database. Some samples were discarded due to structure problem such as missing atoms or wrong chemical valences.


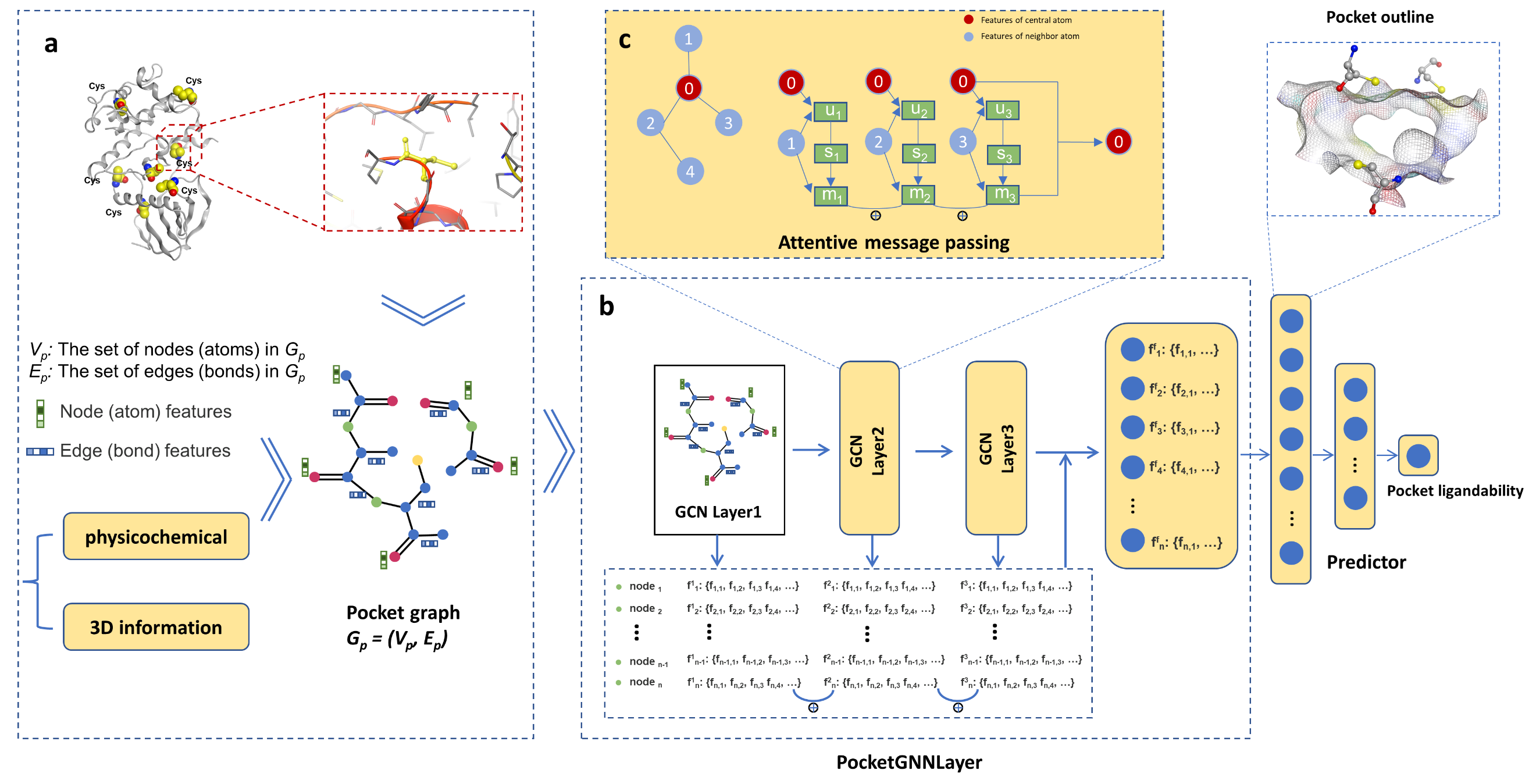


**Figure S2. The workflow of PriDeepCoSI.** (a) The pocket structure of cysteine is transformed into graph $G_{p}$ and embedded with the physicochemical and 3D information. $V_{p}$ and $E_{p}$ denote the set of nodes (atoms) and edges (bonds) in $G_{p}$ respectively. (b) PocketGNNLayer is the main component in PriDeepCoSI to pass message through bonds and get the final state of atoms (to update the properties of central atoms based on the influence of surrounding atoms). The final node features were generated after summarizing the outputs from three GCN layers. (c) Attentive message passing: GCN layers perform neighboring information aggregation and atom hidden state update with an attentive mechanism (to assign different weights to neighbor atoms when their message is transferred to central atom). Finally, these node features were integrated into a vector to represent the properties of the entire pocket (pocket outline) and used for the subsequent prediction of pocket ligandability (represented by the probability value output by model). (see **Section 3.3** of article for details)


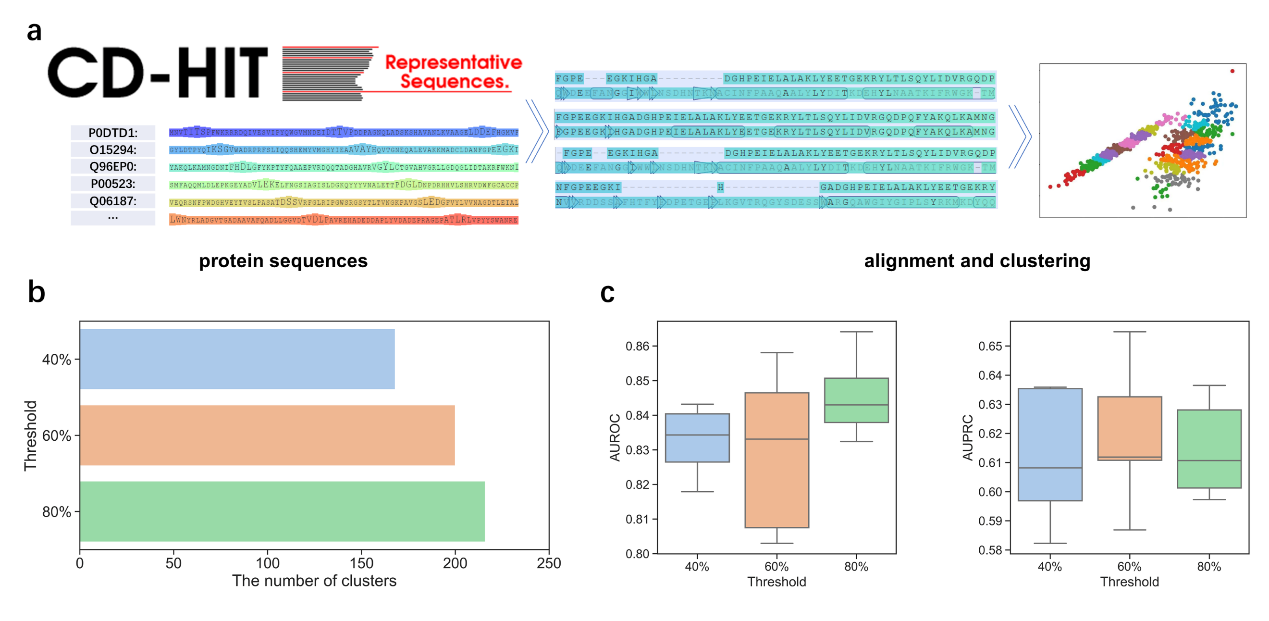


**Figure S3. Dataset splitting based on clustering result.** In order to maximize the diversity between the datasets for model training and evaluation, we clustered the proteins based on their sequences with cd-hit51 before splitting. Different identity values for the clustering thresholds (40%, 60% and 80%) were used to explore the robustness of the model. (a) cd-hit performs sequence alignment and clustering. (b) The number of clusters with different thresholds. (c) The performance of PriDeepCoSI with different clustering threshold.

**The effect of the pocket size on PriDeepCoSI：**

A larger pocket was supposed to provide more information to the model but also might reduce the density of effective knowledge and introduce noise^1, 2^. Thus, the influence of the pocket size on predictive accuracy was further explored with the sizes of the pockets set to 10 Å, 15 Å and 20 Å (**Supporting Information Table S2**). The results showed that the complexity of the graph increased (more edges and nodes) as the pocket became larger. And it took longer for training one epoch (an epoch refers to one cycle through the full training dataset) and the convergence of the training was also delayed (**Supporting Information Figure S4**). Besides, the number of the epochs required for reaching a AUROC value of 0.98 increased with the size of the pocket. Of note, increasing the pocket size did not necessarily lead to better performance especially for the size of 20 Å, which achieved ordinary performance in both the evaluation metrics (**Supporting Information Figure S4a**). AUPRC was used as the criterion for evaluating the accuracy of the model due to the imbalance of the dataset, where 15 Å was found to be the best size and utilized for the subsequent study.


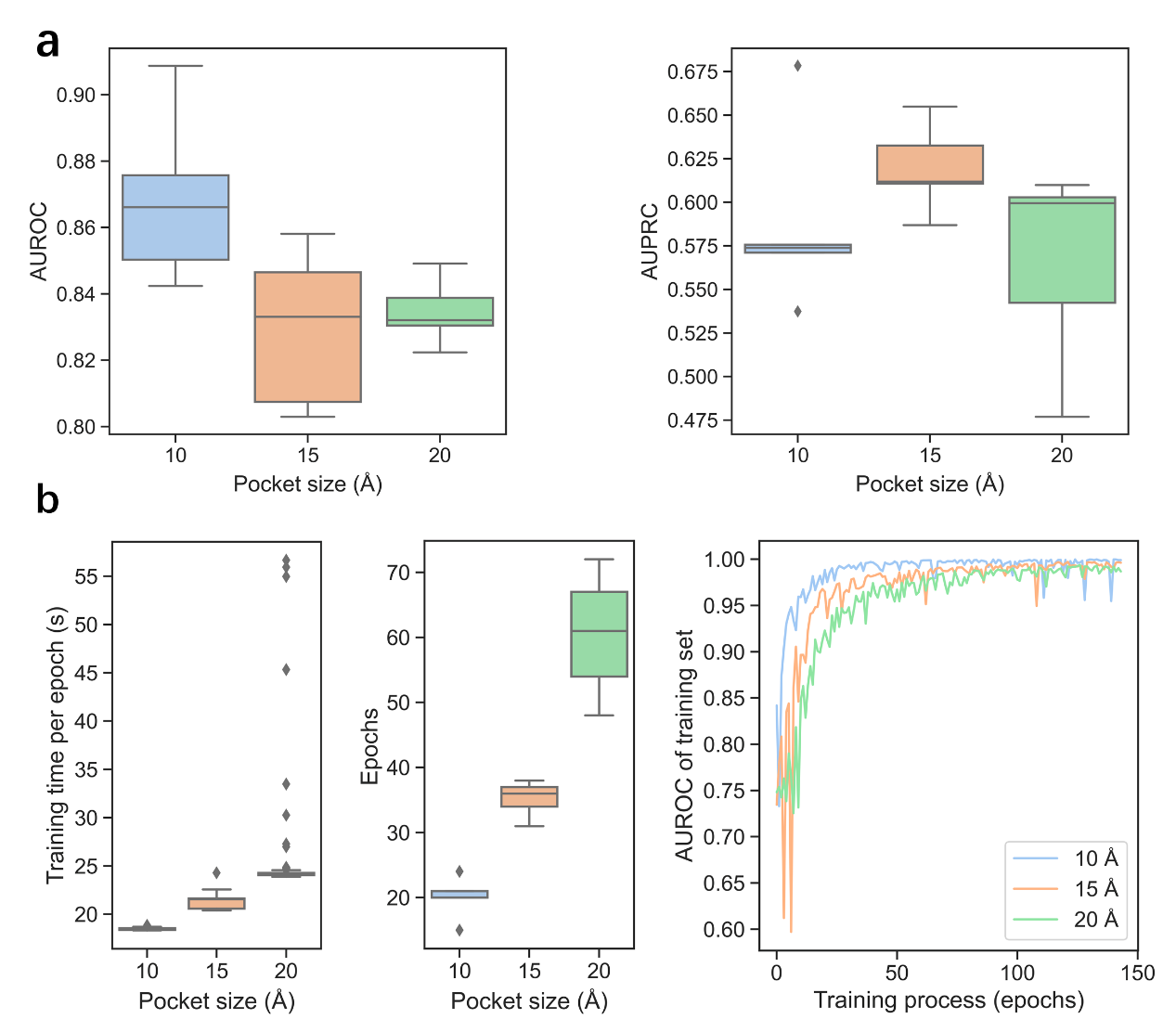


**Figure S4.** **The effect of the pocket size on PriDeepCoSI.** (a) The performance of PriDeepCoSI with different pocket size. (b left) The training time needed for each epoch with different pocket size. (b middle) The number of epochs required for the AUROC of the training set to reach 0.98. (b right) The training process with different pocket size.

**Table S1.** The performance of PriDeepCoSI with different clustering thresholds.

| Threshold | Run_repeat | AUROC | AUPRC |
| --- | --- | --- | --- |
| 40% | 1 | 0.8404 | 0.6082 |
| 40% | 2 | 0.8343 | 0.5969 |
| 40% | 3 | 0.8265 | 0.5823 |
| 40% | 4 | 0.8432 | 0.6354 |
| 40% | 5 | 0.8179 | 0.6359 |
| 40% | average | 0.8325±0.01 | 0.6117±0.02 |
| 60% | 1 | 0.8465 | 0.6549 |
| 60% | 2 | 0.8075 | 0.5869 |
| 60% | 3 | 0.8030 | 0.6119 |
| 60% | 4 | 0.8581 | 0.6326 |
| 60% | 5 | 0.8331 | 0.6108 |
| 60% | average | 0.8296±0.02 | 0.6194±0.03 |
| 80% | 1 | 0.8507 | 0.6107 |
| 80% | 2 | 0.8379 | 0.6013 |
| 80% | 3 | 0.8641 | 0.6281 |
| 80% | 4 | 0.8430 | 0.6365 |
| 80% | 5 | 0.8324 | 0.5973 |
| 80% | average | 0.8456±0.01 | 0.6148±0.02 |

**Table S2.** The performance of PriDeepCoSI with different pocket size.

| Pocket size | Run_repeat | AUROC | AUPRC |
| --- | --- | --- | --- |
| 10 Å | 1 | 0.9087 | 0.6783 |
| 10 Å | 2 | 0.8424 | 0.5757 |
| 10 Å | 3 | 0.8661 | 0.5711 |
| 10 Å | 4 | 0.8503 | 0.5374 |
| 10 Å | 5 | 0.8757 | 0.5739 |
| 10 Å | average | 0.8686±0.03 | 0.5872±0.05 |
| 15 Å | 1 | 0.8465 | 0.6549 |
| 15 Å | 2 | 0.8075 | 0.5869 |
| 15 Å | 3 | 0.803 | 0.6119 |
| 15 Å | 4 | 0.8581 | 0.6326 |
| 15 Å | 5 | 0.8331 | 0.6108 |
| 15 Å | average | 0.8296±0.02 | 0.6194±0.03 |
| 20 Å | 1 | 0.8491 | 0.6099 |
| 20 Å | 2 | 0.8388 | 0.5424 |
| 20 Å | 3 | 0.8321 | 0.6029 |
| 20 Å | 4 | 0.8305 | 0.5995 |
| 20 Å | 5 | 0.8224 | 0.477 |
| 20 Å | average | 0.8346±0.01 | 0.5663±0.06 |

**Table S3.** The performance comparison between DeepCoSI and PriDeepCoSI.

| Model | Run_repeat | AUROC | AUPRC |
| --- | --- | --- | --- |
| PriDeepCoSI | 1 | 0.8465 | 0.6549 |
| PriDeepCoSI | 2 | 0.8075 | 0.5869 |
| PriDeepCoSI | 3 | 0.803 | 0.6119 |
| PriDeepCoSI | 4 | 0.8581 | 0.6326 |
| PriDeepCoSI | 5 | 0.8331 | 0.6108 |
| PriDeepCoSI | average | 0.8296±0.02 | 0.6194±0.03 |
| DeepDoSI | 1 | 0.9183 | 0.7684 |
| DeepDoSI | 2 | 0.9361 | 0.7706 |
| DeepDoSI | 3 | 0.9092 | 0.733 |
| DeepDoSI | 4 | 0.926 | 0.781 |
| DeepDoSI | 5 | 0.9075 | 0.754 |
| DeepDoSI | average | 0.9194±0.01 | 0.7614±0.02 |

**Table S4.** The performance of DeepCoSI with different interaction thresholds.

| Interaction threshold | Run_repeat | AUROC | AUPRC |
| --- | --- | --- | --- |
| 5 Å | 1 | 0.9151 | 0.7155 |
| 5 Å | 2 | 0.8946 | 0.7054 |
| 5 Å | 3 | 0.8942 | 0.6046 |
| 5 Å | 4 | 0.9084 | 0.7017 |
| 5 Å | 5 | 0.9109 | 0.7281 |
| 5 Å | average | 0.9046±0.01 | 0.6911±0.05 |
| 7 Å | 1 | 0.9183 | 0.7684 |
| 7 Å | 2 | 0.9361 | 0.7706 |
| 7 Å | 3 | 0.9092 | 0.733 |
| 7 Å | 4 | 0.926 | 0.781 |
| 7 Å | 5 | 0.9075 | 0.754 |
| 7 Å | average | 0.9194±0.01 | 0.7614±0.02 |
| 10 Å | 1 | 0.913 | 0.7608 |
| 10 Å | 2 | 0.9287 | 0.7527 |
| 10 Å | 3 | 0.9053 | 0.7283 |
| 10 Å | 4 | 0.9103 | 0.7409 |
| 10 Å | 5 | 0.9188 | 0.7184 |
| 10 Å | average | 0.9152±0.01 | 0.7402±0.02 |

**Table S5.** The performance comparison between DeepCoSI and SVM model.

| Model | Run_repeat | AUROC | AUPRC |
| --- | --- | --- | --- |
| DeepDoSI | 1 | 0.8452 | 0.7061 |
| DeepDoSI | 2 | 0.9059 | 0.8296 |
| DeepDoSI | 3 | 0.9321 | 0.8497 |
| DeepDoSI | 4 | 0.8609 | 0.7803 |
| DeepDoSI | 5 | 0.9533 | 0.9114 |
| DeepDoSI | 6 | 0.9038 | 0.7633 |
| DeepDoSI | 7 | 0.8778 | 0.8126 |
| DeepDoSI | 8 | 0.9029 | 0.8522 |
| DeepDoSI | 9 | 0.8806 | 0.8395 |
| DeepDoSI | 10 | 0.9025 | 0.8297 |
| DeepDoSI | average | 0.8965±0.01 | 0.8174±0.03 |
| SVM | 1 | 0.854921 | 0.629947 |
| SVM | 2 | 0.887295 | 0.726946 |
| SVM | 3 | 0.903844 | 0.765678 |
| SVM | 4 | 0.815359 | 0.60421 |
| SVM | 5 | 0.944655 | 0.852476 |
| SVM | 6 | 0.912201 | 0.67453 |
| SVM | 7 | 0.862593 | 0.687351 |
| SVM | 8 | 0.921619 | 0.850538 |
| SVM | 9 | 0.844855 | 0.690094 |
| SVM | 10 | 0.806688 | 0.574734 |
| SVM | average | 0.8754±0.05 | 0.7057±0.1 |

**
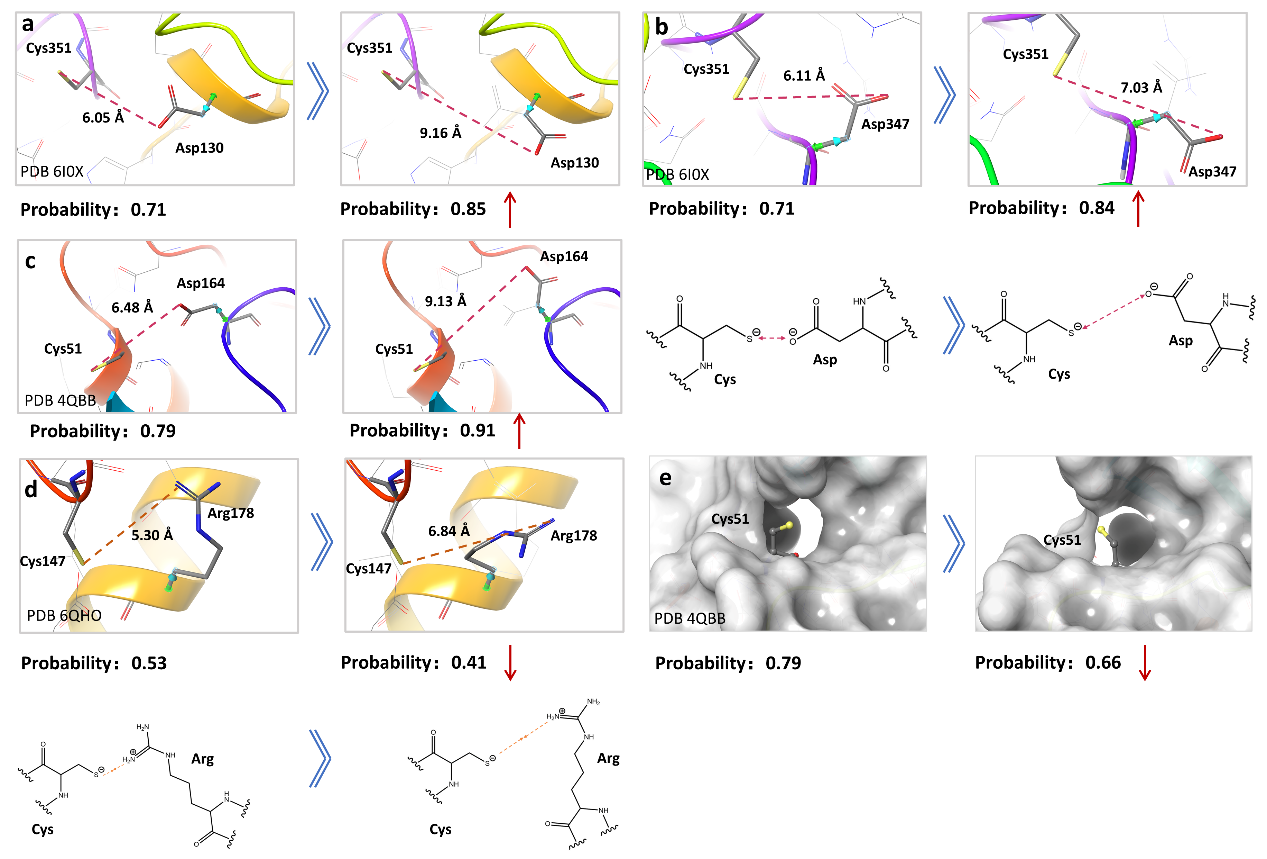
**

**Figure S5.** Changes in the predicted value after the structure modification (Run 1). (a) Structure modification on PDB 6I0X to decrease the electrostatic repulsion between Cys351 and Asp130. (b) Structure modification on PDB 6I0X to decrease the electrostatic repulsion between Cys351 and Asp347. (c) Structure modification on PDB 4QBB to decrease the electrostatic repulsion between Cys51 and Asp164. (d) Structure modification on PDB 6QHO to decrease the electrostatic attraction between Cys147 and Arg178. (e) Structure modification on PDB 4QBB to change the orientation of cysteine from towards the pocket cavity to against the cavity.

**Table S6.** The result of structure modification experiment.

| PDB | Cys | Manipulated residue | Factor | Run_repeat | Probability (before) | Probability (after) | Probability change | As desired? |
| --- | --- | --- | --- | --- | --- | --- | --- | --- |
| 6QHO | Cys147 | Asp277 | Electrostatic repulsion | 1 | 0.53 | 0.68 | 0.15 | Yes |
| 6QHO | Cys147 | Asp277 | Electrostatic repulsion | 2 | 0.51 | 0.61 | 0.1 | Yes |
| 6QHO | Cys147 | Asp277 | Electrostatic repulsion | 3 | 0.53 | 0.63 | 0.1 | Yes |
| 6QHO | Cys147 | Asp277 | Electrostatic repulsion | 4 | 0.5 | 0.64 | 0.14 | Yes |
| 6QHO | Cys147 | Asp277 | Electrostatic repulsion | 5 | 0.48 | 0.63 | 0.15 | Yes |
| 6I0X | Cys351 | Asp347 | Electrostatic repulsion | 1 | 0.71 | 0.84 | 0.13 | Yes |
| 6I0X | Cys351 | Asp347 | Electrostatic repulsion | 2 | 0.54 | 0.73 | 0.19 | Yes |
| 6I0X | Cys351 | Asp347 | Electrostatic repulsion | 3 | 0.82 | 0.95 | 0.13 | Yes |
| 6I0X | Cys351 | Asp347 | Electrostatic repulsion | 4 | 0.71 | 0.79 | 0.08 | Yes |
| 6I0X | Cys351 | Asp347 | Electrostatic repulsion | 5 | 0.91 | 1.02 | 0.11 | Yes |
| 6I0X | Cys351 | Asp130 | Electrostatic repulsion | 1 | 0.71 | 0.85 | 0.14 | Yes |
| 6I0X | Cys351 | Asp130 | Electrostatic repulsion | 2 | 0.54 | 0.59 | 0.05 | Yes |
| 6I0X | Cys351 | Asp130 | Electrostatic repulsion | 3 | 0.82 | 0.93 | 0.11 | Yes |
| 6I0X | Cys351 | Asp130 | Electrostatic repulsion | 4 | 0.71 | 0.81 | 0.1 | Yes |
| 6I0X | Cys351 | Asp130 | Electrostatic repulsion | 5 | 0.91 | 0.98 | 0.07 | Yes |
| 4QBB | Cys51 | Asp164 | Electrostatic repulsion | 1 | 0.79 | 0.91 | 0.12 | Yes |
| 4QBB | Cys51 | Asp164 | Electrostatic repulsion | 2 | 0.68 | 0.8 | 0.12 | Yes |
| 4QBB | Cys51 | Asp164 | Electrostatic repulsion | 3 | 0.77 | 0.97 | 0.2 | Yes |
| 4QBB | Cys51 | Asp164 | Electrostatic repulsion | 4 | 0.68 | 0.8 | 0.12 | Yes |
| 4QBB | Cys51 | Asp164 | Electrostatic repulsion | 5 | 0.82 | 0.98 | 0.16 | Yes |
| 6QHO | Cys147 | Lys165 | Electrostatic attraction | 1 | 0.53 | 0.21 | -0.32 | Yes |
| 6QHO | Cys147 | Lys165 | Electrostatic attraction | 2 | 0.51 | 0.36 | -0.15 | Yes |
| 6QHO | Cys147 | Lys165 | Electrostatic attraction | 3 | 0.53 | 0.26 | -0.27 | Yes |
| 6QHO | Cys147 | Lys165 | Electrostatic attraction | 4 | 0.5 | 0.37 | -0.13 | Yes |
| 6QHO | Cys147 | Lys165 | Electrostatic attraction | 5 | 0.48 | 0.3 | -0.18 | Yes |
| 6QHO | Cys147 | Arg178 | Electrostatic attraction | 1 | 0.53 | 0.41 | -0.12 | Yes |
| 6QHO | Cys147 | Arg178 | Electrostatic attraction | 2 | 0.51 | 0.3 | -0.21 | Yes |
| 6QHO | Cys147 | Arg178 | Electrostatic attraction | 3 | 0.53 | 0.42 | -0.11 | Yes |
| 6QHO | Cys147 | Arg178 | Electrostatic attraction | 4 | 0.5 | 0.39 | -0.11 | Yes |
| 6QHO | Cys147 | Arg178 | Electrostatic attraction | 5 | 0.48 | 0.33 | -0.15 | Yes |
| 6I0X | Cys351 | Cys351 | Cysteine orientation | 1 | 0.71 | 0.41 | -0.3 | Yes |
| 6I0X | Cys351 | Cys351 | Cysteine orientation | 2 | 0.54 | 0.32 | -0.22 | Yes |
| 6I0X | Cys351 | Cys351 | Cysteine orientation | 3 | 0.82 | 0.6 | -0.22 | Yes |
| 6I0X | Cys351 | Cys351 | Cysteine orientation | 4 | 0.71 | 0.5 | -0.21 | Yes |
| 6I0X | Cys351 | Cys351 | Cysteine orientation | 5 | 0.91 | 0.71 | -0.2 | Yes |
| 4QBB | Cys51 | Cys51 | Cysteine orientation | 1 | 0.79 | 0.66 | -0.13 | Yes |
| 4QBB | Cys51 | Cys51 | Cysteine orientation | 2 | 0.68 | 0.6 | -0.08 | Yes |
| 4QBB | Cys51 | Cys51 | Cysteine orientation | 3 | 0.77 | 0.65 | -0.12 | Yes |
| 4QBB | Cys51 | Cys51 | Cysteine orientation | 4 | 0.68 | 0.58 | -0.1 | Yes |
| 4QBB | Cys51 | Cys51 | Cysteine orientation | 5 | 0.82 | 0.71 | -0.11 | Yes |

**Table S7.** The ability of DeepCoSI to identity ligandable Cys on the external test set1.

| PDB | Cys(chain-position) | Number of Cys | Ranking based on predicted probability | Normalized ranking by the number of Cys |
| --- | --- | --- | --- | --- |
| 6IM5 | A-359 | 6 | 1 | 0.167 |
| 2DYL | A-218 | 7 | 3 | 0.429 |
| 5Z1D | A-218 | 6 | 2 | 0.333 |
| 6QFL | A-218 | 8 | 2 | 0.250 |
| 6QFR | A-218 | 6 | 2 | 0.333 |
| 6YFZ | A-218 | 8 | 3 | 0.375 |
| 6YG0 | A-218 | 6 | 2 | 0.333 |
| 6YG1 | A-218 | 7 | 3 | 0.429 |
| 6YZ4 | A-218 | 7 | 3 | 0.429 |
| 2JAV | A-22 | 5 | 1 | 0.200 |
| 2W5A | A-22 | 5 | 1 | 0.200 |
| 2W5B | A-22 | 4 | 1 | 0.250 |
| 2W5H | A-22 | 5 | 1 | 0.200 |
| 2WQO | A-22 | 5 | 1 | 0.200 |
| 2XK3 | A-22 | 5 | 1 | 0.200 |
| 2XK4 | A-22 | 5 | 1 | 0.200 |
| 2XK6 | A-22 | 5 | 1 | 0.200 |
| 2XK7 | A-22 | 5 | 1 | 0.200 |
| 2XK8 | A-22 | 5 | 1 | 0.200 |
| 2XKD | A-22 | 5 | 1 | 0.200 |
| 2XKE | A-22 | 5 | 1 | 0.200 |
| 2XKF | A-22 | 5 | 1 | 0.200 |
| 2XNM | A-22 | 5 | 1 | 0.200 |
| 2XNO | A-22 | 5 | 1 | 0.200 |
| 2XNP | A-22 | 5 | 2 | 0.400 |
| 4A4X | A-22 | 4 | 2 | 0.500 |
| 5M51 | A-22 | 5 | 1 | 0.200 |
| 5M53 | A-22 | 5 | 1 | 0.200 |
| 5M55 | A-22 | 5 | 1 | 0.200 |
| 5M57 | A-22 | 5 | 1 | 0.200 |
| 6SGI | A-22 | 5 | 1 | 0.200 |
| 6SGK | A-22 | 5 | 1 | 0.200 |
| 6SK9 | A-22 | 5 | 2 | 0.400 |
| 3SXR | A-496 | 7 | 2 | 0.286 |
| 3SXS | A-496 | 7 | 1 | 0.143 |
| 4XFQ | A-144 | 8 | 1 | 0.125 |
| 4YTB | A-351 | 7 | 1 | 0.143 |
| 5AK7 | A-351 | 7 | 1 | 0.143 |
| 5DQ8 | A-380 | 4 | 1 | 0.250 |
| 5DQE | A-380 | 4 | 1 | 0.250 |
| 6CDY | A-380 | 4 | 1 | 0.250 |
| 6S64 | A-380 | 4 | 1 | 0.250 |
| 6S66 | A-380 | 4 | 1 | 0.250 |
| 6S69 | A-380 | 4 | 1 | 0.250 |
| 6S6J | A-380 | 4 | 1 | 0.250 |
| 6UYB | A-380 | 4 | 1 | 0.250 |
| 6UYC | A-380 | 4 | 1 | 0.250 |
| 6VAH | A-380 | 4 | 1 | 0.250 |
| 1AZV | A-111 | 4 | 1 | 0.250 |
| 1HL4 | A-111 | 4 | 1 | 0.250 |
| 1OEZ | W-111 | 4 | 1 | 0.250 |
| 1PU0 | A-111 | 4 | 1 | 0.250 |
| 1SPD | A-111 | 4 | 1 | 0.250 |
| 1UXL | A-111 | 4 | 1 | 0.250 |
| 1UXM | A-111 | 4 | 1 | 0.250 |
| 2C9U | A-111 | 4 | 1 | 0.250 |
| 2NNX | A-111 | 4 | 2 | 0.500 |
| 2V0A | A-111 | 4 | 1 | 0.250 |
| 2VR6 | A-111 | 4 | 1 | 0.250 |
| 2VR7 | A-111 | 4 | 1 | 0.250 |
| 2WKO | A-111 | 4 | 1 | 0.250 |
| 2WYT | A-111 | 4 | 1 | 0.250 |
| 2WZ0 | A-111 | 4 | 1 | 0.250 |
| 2WZ5 | A-111 | 4 | 1 | 0.250 |
| 2WZ6 | A-111 | 4 | 1 | 0.250 |
| 2ZKW | A-111 | 4 | 1 | 0.250 |
| 2ZKY | A-111 | 4 | 1 | 0.250 |
| 3CQP | A-111 | 4 | 1 | 0.250 |
| 3ECU | A-111 | 4 | 1 | 0.250 |
| 3ECV | A-111 | 4 | 1 | 0.250 |
| 3ECW | A-111 | 4 | 1 | 0.250 |
| 3GQF | A-111 | 4 | 1 | 0.250 |
| 3GZO | A-111 | 4 | 2 | 0.500 |
| 3GZQ | A-111 | 4 | 1 | 0.250 |
| 3H2P | A-111 | 4 | 1 | 0.250 |
| 3H2Q | A-111 | 4 | 1 | 0.250 |
| 3K91 | A-111 | 4 | 1 | 0.250 |
| 3LTV | A-111 | 4 | 1 | 0.250 |
| 4A7G | A-111 | 4 | 1 | 0.250 |
| 4A7Q | A-111 | 4 | 1 | 0.250 |
| 4A7S | A-111 | 4 | 1 | 0.250 |
| 4A7U | A-111 | 4 | 1 | 0.250 |
| 4A7V | A-111 | 4 | 1 | 0.250 |
| 4B3E | A-111 | 4 | 1 | 0.250 |
| 5K02 | A-111 | 4 | 1 | 0.250 |
| 5O40 | A-111 | 4 | 1 | 0.250 |
| 5U9M | A-111 | 4 | 3 | 0.750 |
| 5YTO | A-111 | 4 | 1 | 0.250 |
| 5YTU | A-111 | 4 | 1 | 0.250 |
| 5YUL | A-111 | 4 | 1 | 0.250 |
| 6SPA | A-111 | 4 | 1 | 0.250 |
| 6SPH | A-111 | 4 | 1 | 0.250 |
| 4GPQ | A-329 | 6 | 1 | 0.167 |
| 4GQ3 | A-329 | 7 | 2 | 0.286 |
| 4GQ6 | A-329 | 7 | 2 | 0.286 |
| 4OG3 | A-329 | 7 | 2 | 0.286 |
| 4OG4 | A-329 | 7 | 2 | 0.286 |
| 4OG5 | A-329 | 7 | 2 | 0.286 |
| 4OG6 | A-329 | 7 | 2 | 0.286 |
| 4OG7 | A-329 | 7 | 2 | 0.286 |
| 4OG8 | A-329 | 7 | 2 | 0.286 |
| 4X5Y | A-329 | 7 | 2 | 0.286 |
| 4X5Z | A-329 | 7 | 2 | 0.286 |
| 5DB0 | A-329 | 7 | 2 | 0.286 |
| 5DB1 | A-329 | 7 | 2 | 0.286 |
| 5DB2 | A-329 | 7 | 2 | 0.286 |
| 5DB3 | A-329 | 7 | 2 | 0.286 |
| 5DD9 | A-329 | 7 | 2 | 0.286 |
| 5DDA | A-329 | 7 | 2 | 0.286 |
| 5DDB | A-329 | 7 | 2 | 0.286 |
| 5DDC | A-329 | 7 | 2 | 0.286 |
| 5DDD | A-329 | 7 | 2 | 0.286 |
| 5DDF | A-329 | 7 | 2 | 0.286 |
| 6BXH | A-329 | 7 | 2 | 0.286 |
| 6BXY | A-329 | 7 | 2 | 0.286 |
| 6BY8 | A-329 | 7 | 2 | 0.286 |
| 6O5I | A-329 | 7 | 2 | 0.286 |
| 6OPJ | A-329 | 7 | 2 | 0.286 |
| 6PKC | A-329 | 6 | 2 | 0.333 |
| 6WNH | A-329 | 7 | 1 | 0.143 |
| 3TLO | A-144 | 11 | 1 | 0.091 |
| 3ZFY | A-717 | 6 | 1 | 0.167 |
| 1I7G | A-275 | 7 | 1 | 0.143 |
| 2NPA | A-275 | 7 | 3 | 0.429 |
| 2P54 | A-275 | 7 | 2 | 0.286 |
| 2REW | A-275 | 7 | 2 | 0.286 |
| 2ZNN | A-275 | 7 | 2 | 0.286 |
| 3FEI | A-275 | 7 | 1 | 0.143 |
| 3G8I | A-275 | 7 | 3 | 0.429 |
| 3KDU | A-275 | 7 | 1 | 0.143 |
| 3SP6 | A-275 | 7 | 2 | 0.286 |
| 3VI8 | A-275 | 7 | 2 | 0.286 |
| 4CI4 | A-275 | 7 | 1 | 0.143 |
| 5HYK | A-275 | 7 | 5 | 0.714 |
| 6KAX | A-275 | 7 | 2 | 0.286 |
| 6KAY | A-275 | 7 | 2 | 0.286 |
| 6KAZ | A-275 | 7 | 2 | 0.286 |
| 6KB0 | A-275 | 7 | 2 | 0.286 |
| 6KB1 | A-275 | 7 | 2 | 0.286 |
| 6KB2 | A-275 | 7 | 2 | 0.286 |
| 6KB3 | A-275 | 7 | 2 | 0.286 |
| 6KB4 | A-275 | 6 | 2 | 0.333 |
| 6KB5 | A-275 | 7 | 2 | 0.286 |
| 6KB6 | A-275 | 7 | 2 | 0.286 |
| 6KB7 | A-275 | 7 | 3 | 0.429 |
| 6KB8 | A-275 | 7 | 2 | 0.286 |
| 6KB9 | A-275 | 7 | 2 | 0.286 |
| 6KBA | A-275 | 7 | 3 | 0.429 |
| 6KXX | A-275 | 7 | 3 | 0.429 |
| 6KXY | A-275 | 7 | 2 | 0.286 |
| 6LX4 | A-275 | 7 | 3 | 0.429 |
| 6LX5 | A-275 | 7 | 2 | 0.286 |
| 6LX6 | A-275 | 7 | 2 | 0.286 |
| 6LX7 | A-275 | 7 | 2 | 0.286 |
| 6LX8 | A-275 | 7 | 2 | 0.286 |
| 6LX9 | A-275 | 7 | 2 | 0.286 |
| 6LXA | A-275 | 7 | 2 | 0.286 |
| 6LXB | A-275 | 7 | 1 | 0.143 |
| 6LXC | A-275 | 7 | 1 | 0.143 |
| 7BPY | A-275 | 7 | 3 | 0.429 |
| 7BPZ | A-275 | 7 | 1 | 0.143 |
| 7BQ0 | A-275 | 7 | 2 | 0.286 |
| 7BQ1 | A-275 | 7 | 2 | 0.286 |
| 7BQ2 | A-275 | 7 | 2 | 0.286 |
| 7BQ3 | A-275 | 7 | 2 | 0.286 |
| 7BQ4 | A-275 | 7 | 2 | 0.286 |
| 5L5A | K-48 | 5 | 1 | 0.200 |
| 4YLU | A-148 | 10 | 1 | 0.100 |
| 1QQP | 3-51 | 4 | 2 | 0.500 |
| 2FD6 | A-13 | 40 | 10 | 0.250 |
| 2I9A | A-13 | 12 | 5 | 0.417 |
| 2FD6 | U-12 | 40 | 3 | 0.075 |
| 4GLY | B-12 | 6 | 2 | 0.333 |
| 4GLY | B-7 | 6 | 1 | 0.167 |
| 6NMB | A-7 | 12 | 6 | 0.500 |
| 3SZA | A-243 | 7 | 1 | 0.143 |
| 4L2O | A-243 | 7 | 1 | 0.143 |
| 4JZ8 | A-242 | 8 | 3 | 0.375 |
| 4JZ9 | A-242 | 8 | 5 | 0.625 |
| 2F1G | A-25 | 9 | 1 | 0.111 |
| 2G7Y | A-25 | 9 | 1 | 0.111 |
| 2H7J | A-25 | 9 | 1 | 0.111 |
| 2HHN | A-25 | 9 | 1 | 0.111 |
| 2R9M | A-25 | 9 | 1 | 0.111 |
| 2R9N | A-25 | 9 | 1 | 0.111 |
| 2R9O | A-25 | 9 | 1 | 0.111 |
| 3N3G | A-25 | 9 | 1 | 0.111 |
| 4P6E | A-25 | 9 | 1 | 0.111 |
| 4P6G | A-25 | 9 | 1 | 0.111 |
| 1YTN | A-403 | 5 | 2 | 0.400 |
| 1YTW | A-403 | 5 | 2 | 0.400 |
| 2I42 | A-403 | 5 | 2 | 0.400 |
| 3F99 | A-403 | 5 | 2 | 0.400 |
| 3F9A | A-403 | 5 | 2 | 0.400 |
| 3F9B | A-403 | 5 | 1 | 0.200 |
| 3U96 | A-403 | 5 | 2 | 0.400 |
| 4YAA | A-403 | 5 | 2 | 0.400 |
| 4Z6B | A-403 | 5 | 2 | 0.400 |
| 4ZI4 | A-403 | 5 | 2 | 0.400 |
| 4ZN5 | A-403 | 5 | 2 | 0.400 |
| 2R4B | A-803 | 6 | 1 | 0.167 |
| 2F82 | A-117 | 11 | 1 | 0.091 |
| 1KAS | A-163 | 4 | 1 | 0.250 |
| 2GFW | A-163 | 4 | 1 | 0.250 |

**Table S8.** The ability of DeepCoSI to identity ligandable Cys on the external test set2.

| PDB | Cys(chain-position) | Number of Cys | Ranking based on predicted probability | Normalized ranking by the number of Cys |
| --- | --- | --- | --- | --- |
| 1AIU | A-32 | 5 | 2 | 0.400 |
| 3G2G | A-423 | 8 | 6 | 0.750 |
| 1K0M | A-24 | 6 | 1 | 0.167 |
| 1H2V | C-44 | 18 | 1 | 0.056 |
| 5YF4 | A-134 | 8 | 3 | 0.375 |
| 1W4R | A-66 | 8 | 2 | 0.250 |
| 2P0W | A-101 | 6 | 1 | 0.167 |
| 1P5Z | B-45 | 5 | 1 | 0.200 |
| 2W8N | A-340 | 11 | 1 | 0.091 |
| 6Q3W | A-182 | 8 | 3 | 0.375 |
| 3WGE | A-217 | 4 | 1 | 0.250 |
| 4UMX | A-269 | 5 | 1 | 0.200 |
| 5VR8 | A-276 | 12 | 1 | 0.083 |
| 4MZ7 | A-522 | 8 | 2 | 0.250 |
| 6GWJ | K-265 | 9 | 3 | 0.333 |
| 4W82 | A-1558 | 6 | 2 | 0.333 |
| 3IAR | A-75 | 5 | 3 | 0.600 |
| 5CCB | A-209 | 6 | 1 | 0.167 |
| 6XOG | A-214 | 8 | 5 | 0.625 |
| 5I96 | A-308 | 7 | 1 | 0.143 |
| 3EUF | A-162 | 13 | 1 | 0.077 |
| 2C6Q | A-186 | 8 | 1 | 0.125 |
| 2IZZ | A-120 | 4 | 3 | 0.750 |
| 2CY7 | A-74 | 10 | 1 | 0.100 |
| 3O0G | A-157 | 7 | 1 | 0.143 |
| 2JIF | A-175 | 5 | 1 | 0.200 |
| 5X5O | A-22 | 7 | 4 | 0.571 |
| 3COG | A-229 | 10 | 1 | 0.100 |
| 1FPZ | A-39 | 11 | 7 | 0.636 |
| 6DCB | A-419 | 5 | 1 | 0.200 |
| 5UVF | A-50 | 5 | 1 | 0.200 |
| 5TDE | B-764 | 6 | 1 | 0.167 |
| 3FY7 | A-22 | 5 | 1 | 0.200 |
| 2BKA | A-172 | 4 | 2 | 0.500 |
| 6HEI | A-171 | 5 | 1 | 0.200 |
| 1XG5 | A-226 | 7 | 2 | 0.286 |
| 2Q8G | A-71 | 4 | 1 | 0.250 |
| 3FD5 | A-31 | 8 | 1 | 0.125 |
| 4FC7 | A-22 | 6 | 2 | 0.333 |
| 5TEY | A-500 | 5 | 3 | 0.600 |
| 4UDD | A-622 | 4 | 1 | 0.250 |

**
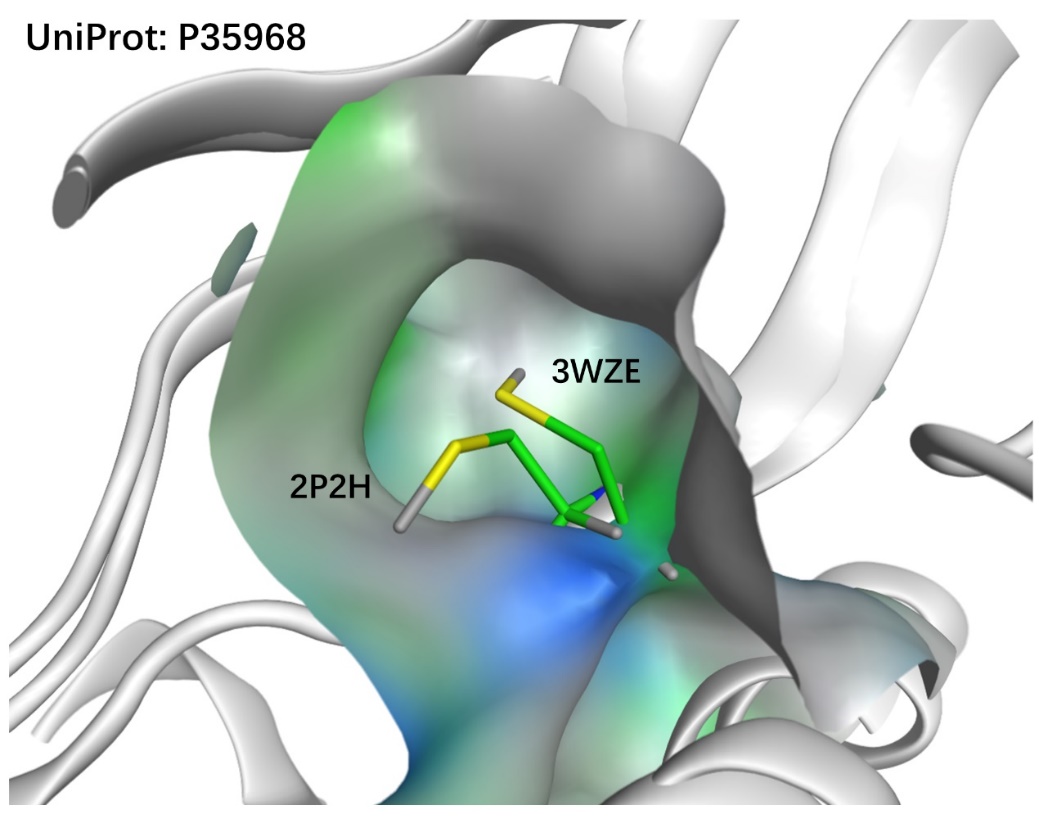
**

**Figure S6.** The superposition of the pocket structures of Cys1045 in VEGFR-2 (RCSB PDB entries: 2P2H, 3WZE)**.** For 2P2H, Cys1045 pointed to the outside of the pocket, which was beneficial to the binding of covalent inhibitors while it pointed to the inside of the pocket for 3WZE, which was unfavorable to the binding.

**Table S9.** Initial atom and bond features.

| Atom Feature | Size | Description | Type |
| --- | --- | --- | --- |
| atom type | 17 | atom type: ['C', 'N', 'O', 'S', 'F', 'P', 'Cl', 'Br', 'I', 'B', 'Si', 'Fe', 'Zn', 'Cu', 'Mn', 'Mo', 'Other'] (one-hot) | physicochemical |
| atom degree | 6 | range: 0-5(one-hot) | physicochemical |
| atom formal charge | 1 | the formal charge of atom | physicochemical |
| number of radical electrons | 1 | the number of radical electrons of atom | physicochemical |
| hybridization | 6 | atom hybridization: ['SP', 'SP2', 'SP3', 'SP3D', 'SP3D2', 'Other'] (one-hot) | physicochemical |
| aromatic | 1 | whether the atom is aromatic | physicochemical |
| number of H | 5 | number of Hs of an atom (range: 0 - 4) (one-hot) | physicochemical |
| charity | 3 | chirality of atom chirality: ['R', 'S', 'Other'] (one-hot) | physicochemical |
| atomic environment vectors | 54 | A vector used to represent the three-dimensional environment of the atom | 3D |

*The physicochemical properties of atoms were calculated by RDKit (Version: 2018.09.3) python packages. Atomic environment vectors (AEV) were calculated by symmetry functions proposed by J. S. Smith, with the TorchANI^3, 4^ (Version: 2.2) python packages. In order to balance the computational efficiency and the information density of AEV, the parameters for AEV calculation were set as follows:

Rcr=12.0, Rca=12.0, EtaR=4.0, ShfR=0.5, EtaA=3.5, Zeta=8.0, ShfA=0, ShtZ=0

| Bond Feature | Size | Description | Type |
| --- | --- | --- | --- |
| bond type | 4 | bond type: ['SINGLE', 'DOUBLE', 'TRIPLE', 'AROMATIC'] (one-hot) | physicochemical |
| conjugation | 1 | whether the bond is conjugated | physicochemical |
| ring | 1 | whether the bond is in a ring of any size | physicochemical |
| stereo | 5 | stereo configuration of a bond: ['STEREONONE', 'STEREOANY', 'STEREOZ', 'STEREOE', 'Other'] (one-hot) | physicochemical |
| angle statistics | 3 | the max, sum and mean values of scaled (multiplied by 0.01) angle between atoms i, j, k | 3D |
| area statistics | 3 | the max, sum and mean values of areas between atoms i, j, k | 3D |
| distance statistics | 3 | the max, sum and mean values of scaled distances (multiplied by 0.1) between atoms i, k | 3D |

*The physicochemical properties of bonds were calculated by RDKit (Version: 2018.09.3) python packages. The 3D properties of bonds were calculated by in-house scripts, according to method proposed by Pengyong Li et al.^5^ (Please go to Github for codes).

**Table S10.** Features for the SVM model^6^.

| Feature | Method | Size |
| --- | --- | --- |
| p*K*_dAve_ | CAVITY 1.1^7^ | 1 |
| p*K*a | PROPKA3^8^ | 1 |
| QSASA | FreeSASA | 1 |
| hDVR | CAVITY 1.1 | 1 |
| hbVR | CAVITY 1.1 | 1 |
| lipVR | CAVITY 1.1 | 1 |
| ENV4A.All | in-house script | 1 |
| ENV6A.All | in-house script | 1 |
| ENV8A.All | in-house script | 1 |
| ENV10A.All | in-house script | 1 |
| Tanaka Descriptors | in-house script | 52 |

**References**

1. Gu, S. K.; Cheng, R.; Jin, Y. C., Feature selection for high-dimensional classification using a competitive swarm optimizer. *SOFT COMPUTING* **2018,** *22* (3), 811-822.

2. Cai, J.; Luo, J. W.; Wang, S. L.; Yang, S., Feature selection in machine learning: A new perspective. *NEUROCOMPUTING* **2018,** *300*, 70-79.

3. Gao, X.; Ramezanghorbani, F.; Isayev, O.; Smith, J. S.; Roitberg, A. E., TorchANI: A Free and Open Source PyTorch-Based Deep Learning Implementation of the ANI Neural Network Potentials. *J Chem Inf Model* **2020,** *60* (7), 3408-3415.

4. Smith, J. S.; Isayev, O.; Roitberg, A. E., ANI-1: an extensible neural network potential with DFT accuracy at force field computational cost. *Chem Sci* **2017,** *8* (4), 3192-3203.

5. Li, P.; Li, Y.; Hsieh, C. Y.; Zhang, S.; Liu, X.; Liu, H.; Song, S.; Yao, X., TrimNet: learning molecular representation from triplet messages for biomedicine. *Brief Bioinform* **2021,** *22* (4).

6. Zhang, W.; Pei, J.; Lai, L., Statistical Analysis and Prediction of Covalent Ligand Targeted Cysteine Residues. *J Chem Inf Model* **2017,** *57* (6), 1453-1460.

7. Yuan, Y.; Pei, J.; Lai, L., Binding site detection and druggability prediction of protein targets for structure-based drug design. *Curr Pharm Des* **2013,** *19* (12), 2326-33.

8. Olsson, M. H.; Søndergaard, C. R.; Rostkowski, M.; Jensen, J. H., PROPKA3: Consistent Treatment of Internal and Surface Residues in Empirical pKa Predictions. *J Chem Theory Comput* **2011,** *7* (2), 525-37.
